# Dynamics of a Cournot game with bounded rational firms and various scale effects

**DOI:** 10.1371/journal.pone.0297275

**Published:** 2024-05-28

**Authors:** Xiaoliang Li, Bo Li, Li Su

**Affiliations:** 1 School of Business, Guangzhou College of Technology and Business, Guangzhou, China; 2 School of Finance, Anhui University of Finance and Economics, Bengbu, China; 3 School of Applied Economics, Renmin University of China, Beijing, China; University of Milano–Bicocca: Universita degli Studi di Milano-Bicocca, ITALY

## Abstract

In this paper, we focus on a dynamic Cournot game in the market with a nonlinear (isoelastic) demand function. In our model, there are *N* competing firms featured by nonlinear cost functions, which enhances our model’s resemblance to real-world scenarios. Firstly, we focus on the homogeneous case where firms’ marginal costs change at equal rates. We establish analytical expressions of the market supply at equilibrium and perform comparative static analysis. In addition, we investigate the local stability under different economies of scale and show that there could be multiple stable equilibria if firms face economies of scale. The heterogeneous case where firms’ marginal costs change at distinct rates is much more complex, thus we investigate the duopoly game with only two firms involved. Methods of symbolic computations such as triangular decomposition and partial cylindrical algebraic decomposition are employed in the analytical investigations of the equilibrium, which is nearly impossible by using the pencil-and-paper approach since the closed-form equilibrium is quite complicated. According to the computational results, we derive that two stable positive equilibria may coexist if both firms face economies of scale. Additionally, we conduct preliminary numerical simulations and find two different types of complex dynamics of the model considered in this paper: complex trajectories such as periodic and chaotic orbits may appear; the topological structure of the basins of attraction may be complex.

## 1 Introduction

The Cournot competition model is a well-known economic model that analyzes the strategic interaction between firms in an oligopoly market. The Cournot-Nash equilibrium [1] has been extensively used in many areas, such as economics, management, sociology, and computer science. Examining the stability of the Cournot-Nash equilibrium is essential: If a model contains an unstable equilibrium, it is difficult to attain in the real economy. Especially, if an economy is initially at an unstable equilibrium, even a small deviation in firms’ behavior or market disruptions due to some external forces, might lead the economy to move away from this equilibrium over time. Many empirical studies reveal that the economic patterns we observe typically align with stable equilibria. In other words, empirical data does not support unstable equilibria.

Furthermore, the comparative static analysis typically assumes that the equilibrium being examined is stable. Therefore, if the stability of the equilibrium cannot be ensured, such an analysis would yield problematic results. Regarding this issue, Dixit [2] comprehensively examines the principles and approaches involved in the comparative static analysis of oligopoly competitions, specifically focusing on the Cournot and Bertrand models. On the other hand, the seminal work by Theocharis [3] focuses on the stability of the Cournot model. He assumes that the market has a linear demand function, recognized by all competing firms, and all firms have linear cost functions. Under these assumptions, Theocharis [3] develops a discrete dynamic game and demonstrates that the equilibrium is stable with no more than three firms. However, the model loses stability with more than three firms.

Following the work of Theocharis [3], Fisher [4], McManus and Quandt [5] improve the demonstration by Theocharis [3] while maintaining the same conclusion. To be specific, Fisher [4], as well as McManus and Quandt [5], extend the work of [3] by retaining the linear demand function but removing the assumption of constant marginal costs by firms. For example, McManus and Quandt [5] follow the dynamic adjustment mechanism the best response functions as in [3], whereas Fisher [4] introduces the bounded rationality and employs the dynamic adjustment mechanism that gradually adjusts their output. In contrast to Theocharis [3], their stability analysis investigates continuous dynamic models. In other words, the model of Theocharis [3] can be viewed as a special case of [4, 5]. Similarly, Sato and Nagatani [6] modify the standard Cournot model by assuming that players make naive conjectures that their rivals would produce the same outputs as in the previous period, and they examine the local stability of their model. Also, Hadar [7] applies a discrete dynamic system to describe the oligopolistic market with differentiated products and derives sufficient but not necessary conditions for local stability. Additionally, Zhang and Zhang [8] propose a model where each firm sells multiple products in several markets. They establish sufficient conditions for the stability of the Cournot-Nash equilibrium and discuss potential applications of these stability findings in the comparative static analysis within their framework.

On the other hand, there is a large number of influential studies on the stability analysis of the continuous Cournot model, including [9–13], to name a few. The stability conditions for the continuous dynamic model are weaker than those for the discrete dynamic model discussed above. For example, Hahn [9] shows that the stability of the continuous model’s equilibrium relies on the appropriate shaping of the demand and marginal cost functions. Okuguchi [10] extends the result of Hahn [9] by giving a weaker stability condition. Similarly, Seade [11] provides a comprehensive analysis of the sufficient conditions for the instability of the equilibrium of the continuous Cournot model. Also, Al-Nowaihi and Levine [12] examine the global stability of the continuous Cournot dynamic model and provide sufficient conditions. They particularly improve Hahn’s arguments, which are also employed by Okuguchi [10]. Finally, Furth [13] discusses the standard Cournot competition, with homogeneous and heterogeneous firms. He considers the local Cournot-Nash equilibrium as the critical point of the Morse-Smale vector field and demonstrates the existence of at least one stable equilibrium using Morse’s inequality.

In recent decades, research on the Cournot game has achieved significant advancement. To name a few, Dastidar [14] focuses on a continuous homogeneous product model with a unique equilibrium. He finds that the unique equilibrium is always locally stable for duopolistic markets. For markets with more than three firms, the stability condition of the equilibrium is also relatively weak. Bischi et al. [15] consider the case where firms only know limited information regarding their profit functions and adjust their output according to signals of marginal revenues. They show that if firms overreact to these signals, the trajectory will not reach the Cournot-Nash equilibrium, and instead result in complex dynamics.

Meanwhile, Ahmed et al. [16] extend these results to the differentiated product market; Agliari et al. [17] introduce an adaptive adjustment mechanism and investigate the possibility of the global bifurcation associated with the subcritical Neimark-Sacker bifurcation, and discover the existence of repelling closed invariant curves; Puu [18] studies oligopoly models in which the firms are constrained by their capacity limit, and show the equilibrium of such models is more stable. In addition, Bischi et al. [19] introduce the concept of the “local monopolistic approximation” (LMA) adjustment mechanism by assuming that firms can make linear approximations of the market demand function. They show that less information might lead to a more stable equilibrium when firms adopt naive conjectures about their rivals. Another issue is how to analyze firms’ possible outcomes using heterogeneous decision mechanisms, with incomplete information on their competitors. In this strand of studies, extensive work has been done by Cavalli and Naimzada [20], Cavalli et al. [21], Tramontana et al. [22], Li and Su [23]. Furthermore, modern tools based on symbolic computations (see, e.g., [24–26] for additional details) have greatly improved analytical studies on the stability of dynamic oligopoly games. Also, many other related studies on the dynamics of oligopoly models should be mentioned, including [27–32].

Our study is an extension of Fisher [4], McManus and Quandt [5]. Specifically, we adopt a similar cost structure (i.e., the quadratic form $e_{i}+c_{i}q_{i}+d_{i}q_{i}^{2}$), but establish a different mechanism for adjusting the output, and perform comparative statics and stability analyses on the model. In contrast to the models of Fisher [4] and McManus and Quandt [5] that adopt linear demand functions, we leverage a nonlinear (isoelastic) demand curve instead. First, we focus on the homogeneous case where firms’ marginal costs change at equal rates, i.e., identical *d*_*i*_. We obtain an analytical solution for the market supply corresponding to the Cournot-Nash equilibrium and derive a sufficient condition for the equilibrium to be locally stable. We find that the equilibrium will be destabilized if the number of firms is sufficiently large, which is consistent with the findings by Fisher [4], McManus and Quandt [5]. Plus, we show the existence of multiple stable equilibria under economies of scale, which is opposite to Fisher [4], McManus and Quandt [5], where only one unique stable equilibrium exists. The existence of multiple equilibria may lead to complex dynamics, which bring challenges to standard theoretical analysis and weaken model prediction. The case of heterogeneous *d*_*i*_ is much more complex, thus we investigate the duopoly game with heterogeneous *d*_*i*_. Methods of symbolic computations such as triangular decomposition and partial cylindrical algebraic decomposition are employed in the analytical investigations of the equilibrium, which is nearly impossible by using the pencil-and-paper approach since the closed-form equilibrium is quite complicated. According to the computational results, we derive that there may exist two stable positive equilibria if both *d*_1_ and *d*_2_ take negative values. Additionally, we conduct numerical simulations and find two different types of complex dynamics of the models considered: complex trajectories such as periodic and chaotic orbits may appear in both the homogeneous and heterogeneous *d*_*i*_ cases; the topological structure of the basins of attraction may be complex in the case of heterogeneous rather than homogeneous *d*_*i*_.

The rest of the paper is organized as follows. In Section 2, we set up the model, introduce the assumption of firms’ bounded rationality, and give a sufficient condition for the stability of model equilibrium. Section 3 discusses the homogeneous case where firms’ marginal costs change at equal rates, and provides a comprehensive analysis, including comparative static and stability analysis. As a comparison, Section 4 discusses the case of duopolistic competition, where firms’ marginal costs change at distinct rates. In Section 5, we conduct numerical simulations to explore the complex dynamics of our model. The final section provides conclusions and possible extensions.

## 2 Model setup

Consider an oligopoly market with *N* (*N* > 1) firms, each producing homogeneous products. The output of firm *i* is denoted by *q*_*i*_, and the total market supply (aggregate production) is $Q=\sum_{i=1}^{N}q_{i}$, so the inverse market demand function (price function) is denoted as *P*(*Q*). In economics, *P*(*Q*) is usually assumed linear for simplicity but may not align with real cases. Here, we follow Puu [33] and assume the market price is the inverse of the aggregate market output, i.e.,

$$P\left(Q\right)=\frac{1}{Q}.$$

That is, the market demand is isoelastic. In addition, we assume that the cost function of firm *i* is quadratic, i.e.,

$$C_{i}\left(q_{i}\right)=e_{i}+c_{i}q_{i}+d_{i}q_{i}^{2},$$

where *e*_*i*_ ≥ 0 is the fixed cost of firm *i*, and its marginal cost is $C_{i}^{\prime }\left(q_{i}\right)=c_{i}+2d_{i}q_{i}$. Here, we assume that *c*_*i*_ > 0. In this context, the value of *d*_*i*_ has important economic implications, i.e., *d*_*i*_ > 0, *d*_*i*_ = 0, and *d*_*i*_ < 0 correspond to increasing (i.e., the diseconomies of scale), constant, and decreasing (i.e., the economies of scale) marginal cost, respectively. Also, we should have *C*_*i*_(*q*_*i*_) > 0 and $C_{i}^{\prime }\left(q_{i}\right)>0$ from an economic perspective. The condition of *C*_*i*_(*q*_*i*_) > 0 can be guaranteed if the fixed cost *e*_*i*_ is sufficiently large. However, the condition of $C_{i}^{\prime }\left(q_{i}\right)>0$ depends on both *c*_*i*_ and *d*_*i*_. For more details, readers can refer to Remark 1.

In the meantime, our model is featured by discrete time, where all firms make dynamic production decisions at the beginning of each period (e.g., every month or every year) to determine their own output (i.e., *q*_*i*_(*t*)) for the current period. Based on our assumptions, the profit of firm *i* at period *t* is

$$\Pi_{i}\left(t\right)=P\left(Q\left(t\right)\right)q_{i}\left(t\right)-C_{i}\left(q_{i}\left(t\right)\right)=\frac{q_{i}\left(t\right)}{Q(t)}-e_{i}-c_{i}q_{i}\left(t\right)-d_{i}q_{i}^{2}\left(t\right).$$

In the previous literature, economists usually employ static models, where companies are essentially assumed to know in advance the above profit functions and maximize their profits accordingly. However, firms in these static models suffer from two inappropriate assumptions, i.e., perfect information about their rivals and no uncertainties about the market demand. However, in reality, the following assumptions are more reasonable.

1. Firms cannot predict their rivals’ exact output; as a result, they cannot predict the market supply *Q*(*t*) in advance.
2. Market demand is usually uncertain and firms cannot compute the exact demand function, especially when the market demand is nonlinear upon prices.

In the following, we discuss these two points separately and set up bounded rationality for firms in an oligopoly model.

First, we assume that firms can guess or estimate their rivals’ output, and use $q_{i}^{e}\left(t\right)$ to denote the estimates of firm *i*’s output at period *t*, then firm *i*’s estimates of the total market supply at period *t* becomes $Q_{i}^{e}\left(t\right)=q_{i}\left(t\right)+\sum_{j\neq i}q_{j}^{e}\left(t\right)$, where *q*_*i*_(*t*) is the exact rather than estimated value of the output since firm *i* knows its own production schedule. Previous literature (see [15, 19–21] among others) adopts the naive expectation assumption, namely $q_{j}^{e}\left(t\right)=q_{j}\left(t-1\right)$. That is, a firm assumes that its opponents’ output in the current period remains the same as in the previous period. If the market demand function is known, firm *i* can estimate its profit at period *t* as

$$\Pi_{i}^{e}\left(t\right)=P\left(Q_{i}^{e}\left(t\right)\right)q_{i}\left(t\right)-C_{i}\left(q_{i}\left(t\right)\right),$$

where

$$Q_{i}^{e}\left(t\right)=q_{i}\left(t\right)+\sum_{j\neq i}q_{j}\left(t-1\right).$$

The firm then chooses its optimal output at period *t* by maximizing its estimated profit $\Pi_{i}^{e}\left(t\right)$, i.e.,

$$q_{i}\left(t\right)=\text{arg}\;\underset{q_{i}}{\text{max}}\;\Pi_{i}^{e}\left(t\right)=\text{arg}\;\underset{q_{i}}{\text{max}}\;\left[P\left(Q_{i}^{e}\left(t\right)\right)q_{i}\left(t\right)-C_{i}\left(q_{i}\left(t\right)\right)\right].$$

The first-order condition for the above optimization problem is

$$\frac{\partial \Pi_{i}^{e}\left(t\right)}{\partial q_{i}\left(t\right)}=\frac{1}{Q_{i}^{e}\left(t\right)}-\frac{q_{i}\left(t\right)}{{\left(Q_{i}^{e}\left(t\right)\right)}^{2}}-c_{i}-2d_{i}q_{i}\left(t\right)=0,\;\;\;i=1,\ldots ,N.$$

The second point, as discussed above, implies that even if a firm can obtain the production plans of its rivals in advance, and then know the market supply, it cannot calculate its profit precisely and maximize its profit due to uncertain market demand. Instead, Bischi et al. [19] and Tuinstra [34] propose a reasonable assumption, which can be traced back to Silvestre [35]. Aligned with them, we assume that firms perform the so-called “local monopolistic approximation” (or LMA for short). Specifically, we assume that firm *i* can observe the market price *p*(*t* − 1) in the last period and the corresponding market supply *Q*(*t* − 1), and can get the slope of the market inverse demand function around (*p*(*t* − 1), *Q*(*t* − 1)), i.e., *P*′(*Q*(*t* − 1)). The slope of the inverse demand function can be computed through market surveys and business experiments, which are widely adopted by firms recently. Therefore, this assumption is appropriate. Then, firm *i* can make a linear approximation of the market price at period *t* to be

$$p_{i}^{e}\left(t\right)=P\left(Q\left(t-1\right)\right)+P^{\prime }\left(Q\left(t-1\right)\right)\left(Q_{i}^{e}\left(t\right)-Q\left(t-1\right)\right).$$

Here $Q_{i}^{e}\left(t\right)=q_{i}\left(t\right)+\sum_{j\neq i}q_{j}^{e}\left(t\right)$ is firm *i*’s estimation of the market supply for period *t*, which also requires expected output of its rivals. Since we assume that $q_{j}^{e}\left(t\right)=q_{j}\left(t-1\right)$, we have

$$p_{i}^{e}\left(t\right)=\frac{1}{Q(t-1)}-\frac{1}{Q^{2}\left(t-1\right)}(q_{i}\left(t\right)-q_{i}\left(t-1\right)).$$

Accordingly, the predicted profit of firm *i* in period *t* is:

$$\Pi_{i}^{e}\left(t\right)=q_{i}\left(t\right)[\frac{1}{Q(t-1)}-\frac{1}{Q^{2}\left(t-1\right)}(q_{i}\left(t\right)-q_{i}\left(t-1\right))]-e_{i}-c_{i}q_{i}\left(t\right)-d_{i}q_{i}^{2}\left(t\right).$$

By solving the first-order condition, the best response function of firm *i* is

$$q_{i}\left(t\right)=\frac{1}{2}(\frac{q_{i}\left(t-1\right)+Q\left(t-1\right)-c_{i}Q^{2}\left(t-1\right)}{d_{i}Q^{2}\left(t-1\right)+1}).$$

As a result, each firm’s output dynamics can be derived as follows:

$$\begin{array}{c} \left\{ \begin{array}{cc} q_{1}\left(t+1\right)= & \;\frac{1}{2}(\frac{q_{1}\left(t\right)+Q\left(t\right)-c_{1}Q^{2}\left(t\right)}{d_{1}Q^{2}\left(t\right)+1}),\\ q_{2}\left(t+1\right)= & \;\frac{1}{2}(\frac{q_{2}\left(t\right)+Q\left(t\right)-c_{2}Q^{2}\left(t\right)}{d_{2}Q^{2}\left(t\right)+1}),\\ & \;\vdots \\ q_{N}\left(t+1\right)= & \;\frac{1}{2}(\frac{q_{N}\left(t\right)+Q\left(t\right)-c_{N}Q^{2}\left(t\right)}{d_{N}Q^{2}\left(t\right)+1}). \end{array}\right. \end{array}$$ (1)

The following proposition provides a sufficient condition for the local stability of map (1) provided that its equilibrium is acquired.

**Proposition 1**. *Suppose that* $\left(q_{1}^{*},q_{2}^{*},\ldots ,q_{N}^{*}\right)$ *is the equilibrium of map* (1), *and denote* $Q^{*}=\sum_{i=1}^{N}q_{i}^{*}$. *If for all i* = 1, …, *N*,

$$|1-d_{i}q_{i}^{*}Q^{*}-c_{i}Q^{*}|+\left(N-1\right)|\frac{1}{2}-\frac{d_{i}}{2}(Q^{*}+2q_{i}^{*})Q^{*}-c_{i}Q^{*}|<{\left(1+d_{i}Q^{*2}\right)}^{2},$$

*then* $\left(q_{1}^{*},q_{2}^{*},\ldots ,q_{N}^{*}\right)$ *is locally stable*.

*Proof*. The proof of Proposition 1 is similar to that of Bischi et al. [19], while we leverage the lemma in S1A Appendix of Bischi et al. [19]. Specifically, this lemma reveals an important mathematical feature: if λ is an eigenvalue of the matrix *J* and ‖ ⋅ ‖ represents any norm of a matrix, then |λ| ≤ ‖*J*‖.

To determine the local stability of the equilibrium $\left(q_{1}^{*},q_{2}^{*},\ldots ,q_{N}^{*}\right)$, we need to examine the Jacobian matrix at the equilibrium: if all eigenvalues of the Jacobian matrix have their modulus smaller than 1, then the equilibrium is locally stable; otherwise, if any eigenvalue has its modulus greater than 1, then the equilibrium is unstable. Regarding map (1), we can see that the diagonal elements of its Jacobian matrix *J* are

$$J_{ii}=\frac{1-d_{i}q_{i}^{*}Q^{*}-c_{i}Q^{*}}{{\left(1+d_{i}Q^{*2}\right)}^{2}},$$

and the non-diagonal elements are

$$J_{ij}=\frac{1/2-d_{i}/2(Q^{*}+2q_{i}^{*})Q^{*}-c_{i}Q^{*}}{{\left(1+d_{i}Q^{*2}\right)}^{2}}.$$

We use the ∞-norm of *J*, i.e., ${\left\|J\right\|}_{\infty }={\text{max}}_{i}\;\sum_{j=1}^{N}\left|J_{ij}\right|$. According to the lemma in S1A Appendix of Bischi et al. [19], all the eigenvalues of the Jacobian matrix *J* have their modulus smaller than 1 if and only if for all *i* = 1, …, *N*,

$$|J_{ii}|+\sum_{j\neq i}\left|J_{ij}\right|<1.$$

By plugging in the expressions of *J*_*ii*_ and *J*_*ij*_, we have

$$|\frac{1-d_{i}q_{i}^{*}Q^{*}-c_{i}Q^{*}}{{\left(1+d_{i}Q^{*2}\right)}^{2}}|+\left(N-1\right)|\frac{1/2-d_{i}/2(Q^{*}+2q_{i}^{*})Q^{*}-c_{i}Q^{*}}{{\left(1+d_{i}Q^{*2}\right)}^{2}}|<1,$$

which is equivalent to

$$|1-d_{i}q_{i}^{*}Q^{*}-c_{i}Q^{*}|+\left(N-1\right)|\frac{1}{2}-\frac{d_{i}}{2}(Q^{*}+2q_{i}^{*})Q^{*}-c_{i}Q^{*}|<{\left(1+d_{i}Q^{*2}\right)}^{2}.$$

The proof is completed.

Proposition 1 offers a sufficient condition for an equilibrium of the model to be locally stable. However, its application needs to obtain the values of $q_{1}^{*},q_{2}^{*},\ldots ,q_{N}^{*}$ in advance, preventing us from employing it to determine the local stability of parametric models. This motivates the next section, where we comprehensively study the case with homogeneous *d*_*i*_, and derive results close to the necessary and sufficient conditions for the local stability.

## 3 Homogeneous *d*_*i*_

To simplify our analysis, we consider the case with identical *d*_*i*_ in all firms’ cost functions, i.e., *d*_1_ = *d*_2_ = ⋯ = *d*_*N*_ = *d*. This assumption is the same as in Fisher [4] and in McManus and Quandt [5], which is reasonable when all firms in the market have similar management and technology.

Accordingly, we sum all the equations in (1) and notice that $Q=\sum_{i}^{N}q_{i}$. Thus, we have

$$\begin{array}{c} Q\left(t+1\right)=\frac{1}{2}(\frac{Q\left(t\right)+NQ(t)-CQ^{2}\left(t\right)}{dQ^{2}\left(t\right)+1}), \end{array}$$ (2)

where $C=\sum_{i=1}^{N}c_{i}>0$. The above system is called the *aggregate map* that describes the dynamics of the market supply *Q*. Thus, we transform the *N*-dimensional system (1) into a simpler system (2) with only one dimension. This transformation greatly simplifies the theoretical derivation process.

According to map (1), we know

$$\frac{q_{i}\left(t+1\right)}{q_{j}\left(t+1\right)}=\frac{q_{i}\left(t\right)+Q\left(t\right)-c_{i}Q^{2}\left(t\right)}{q_{j}\left(t\right)+Q\left(t\right)-c_{i}Q^{2}\left(t\right)}.$$

By summing up the above equation for *i* = 1, …, *N*, we get that

$$\begin{array}{c} \frac{Q(t+1)}{q_{j}\left(t+1\right)}=\frac{Q\left(t\right)+NQ\left(t\right)-CQ^{2}\left(t\right)}{q_{j}\left(t\right)+Q\left(t\right)-c_{j}Q^{2}\left(t\right)}. \end{array}$$ (3)

Thus, the investigation of the dynamic behavior of maps (1) and (2) is essentially equivalent. In particular, regarding the stability analysis, the stability of the equilibrium of system (2) is equivalent to that of system (1).

Let *Q*(*t* + 1) = *Q*(*t*) = Q*. Then we obtain the equilibrium equation

$$\begin{array}{c} Q^{*}=\frac{1}{2}(\frac{Q^{*}+NQ^{*}-CQ^{*2}}{dQ^{*2}+1}). \end{array}$$ (4)

We can obtain the market supply at the equilibrium by solving this equation. Let $\left(q_{1}^{*},q_{2}^{*},\ldots ,q_{N}^{*}\right)$ be the equilibrium of map (1). Accordingly, we have

$$\frac{q_{i}^{*}}{q_{j}^{*}}=\frac{q_{i}^{*}+Q^{*}-c_{i}Q^{*2}}{q_{j}^{*}+Q^{*}-c_{j}Q^{*2}},$$

which implies that

$$\begin{array}{c} \frac{q_{i}^{*}}{q_{j}^{*}}=\frac{Q^{*}-c_{i}Q^{*2}}{Q^{*}-c_{j}Q^{*2}}=\frac{1-c_{i}Q^{*}}{1-c_{j}Q^{*}}. \end{array}$$ (5)

It can be derived that $q_{i}^{*}>q_{j}^{*}$ if *c*_*i*_ < *c*_*j*_. That is, a higher initial marginal cost leads to less output, i.e., less productive firms that produce less efficiently get smaller market shares. This is consistent with the allocation efficiency in an economy. Furthermore, by summing up (5) for *i* = 1, 2, …, *N*, we have

$$\frac{Q^{*}}{q_{j}^{*}}=\frac{N-CQ^{*}}{1-c_{j}Q^{*}}.$$

Therefore, we have

$$\begin{array}{c} q_{j}^{*}=\frac{Q^{*}-c_{j}Q^{*2}}{N-CQ^{*}}. \end{array}$$ (6)

Consequently, firm *i*’s output at the equilibrium (i.e., $q_{j}^{*}$) can be directly recovered from the equilibrium of the market supply Q*.

First, we assume *d* ≠ 0 and leave the special case of *d* = 0 for the end of this section. If *d* ≠ 0, from (4), we can obtain three equilibria of Q* in closed-form, i.e.,

$$\begin{array}{c} Q_{1}^{*}=0,\\ Q_{2}^{*}=-\frac{C+\sqrt{C^{2}+8dN-8d}}{4d},\\ Q_{3}^{*}=-\frac{C-\sqrt{C^{2}+8dN-8d}}{4d}. \end{array}$$

The first equilibrium $Q_{1}^{*}=0$ of map (2) corresponds to the zero equilibrium, i.e., $\left(q_{1}^{*},q_{2}^{*},\ldots ,q_{N}^{*}\right)=\left(0,0,\ldots ,0\right)$ of map (1), which is trivial. Since *N* ≥ 2, we know that diseconomies of scale (i.e., *d* > 0) imply *C*^2^ + 8*dN* − 8*d* > 0, and thus both $Q_{2}^{*}$ and $Q_{3}^{*}$ are real solutions. It is evident that the equilibrium corresponding to $Q_{2}^{*}<0$ is not practical in economics. In contrast, $Q_{3}^{*}>0$ always holds. Therefore, we should focus on the last equilibrium when considering diseconomies of scale. In contrast, with economies of scale (i.e., *d* < 0), if *C*^2^ + 8*dN* − 8*d* ≥ 0, then one can see that both $Q_{2}^{*}$ and $Q_{3}^{*}$ are real solutions with $Q_{2}^{*}>0$ and $Q_{3}^{*}>0$. Accordingly, we have the following theorem.

**Theorem 1**. *Under diseconomies of scale* (*d* > 0), *map* (1) *always possesses three equilibria* $\left(q_{1}^{*},q_{2}^{*},\ldots ,q_{N}^{*}\right)$, *among which* $Q_{1}^{*}=0$ *and* $Q_{3}^{*}>0$. *Under economies of scale* (*d* < 0), *the number of the equilibria depends on the sign of C*^2^ + 8*dN* − 8*d*. *More precisely*,

- *If C*^2^ + 8*dN* − 8*d* < 0, *there is only one real equilibrium, i.e*., $Q_{1}^{*}=0$;
- *If C*^2^ + 8*dN* − 8*d* = 0, *there are two real equilibria satisfying* $Q_{1}^{*}=0$ *and* $Q_{2}^{*}=Q_{3}^{*}=-C/4d>0$;
- *If C*^2^ + 8*dN* − 8*d* > 0, *there are three real equilibria satisfying* $Q_{1}^{*}=0$, $Q_{2}^{*}>Q_{3}^{*}>0$.

*Remark* 1. From an economic point of view, the equilibria in Theorem 1 should satisfy that $C_{i}^{\prime }\left(q_{i}^{*}\right)=c_{i}+2dq_{i}^{*}>0$. This is evident if *d* ≥ 0 as both $q_{i}^{*}>0$ and *c*_*i*_ > 0 are prerequisites. On the other hand, suppose that *d* < 0 and *C*^2^ + 8*dN* − 8*d* > 0; according to (6), it is required that

$$c_{i}+2dq_{i}^{*}=c_{i}+2d\frac{Q^{*}-c_{i}Q^{*2}}{N-CQ^{*}}>0.$$

For

$$Q_{2}^{*}=-\frac{C+\sqrt{C^{2}+8dN-8d}}{4d},$$

we have

$$\begin{array}{c} c_{i}+2dq_{i}^{*}=\frac{-2d(C+\sqrt{C^{2}+8dN-8d}-2c_{i})}{C^{2}+C\sqrt{C^{2}+8dN-8d}+4dN}. \end{array}$$ (7)

Since *N* ≥ 2 and *C*^2^ + 8*dN* − 8*d* > 0, then *C*^2^ + 4*d* = *C*^2^ + 8*dN* − 8*d* + 4*d*(2 − *N*) > 0. Thus, the denominator of (8) is positive. Furthermore, the numerator of (8) is positive if $C+\sqrt{C^{2}+8dN-8d}-2c_{i}>0$, which is likely to be true if the initial marginal cost *c*_*i*_ of firm *i* is not excessively high.

Then, we focus on the effect of a change in the economic environment on the market supply and the product price via comparative static analysis. For example, how will the market supply and the product price change if a new firm enters? To address this question, we assume that the marginal cost of the new firm is *C*′(*q*) = *b* + 2*dq* (*b* > 0), then the market supply at the unique positive equilibrium with the new entrant becomes

$$Q_{3}^{*}=-\frac{C+b-\sqrt{{\left(C+b\right)}^{2}+8d(N+1)-8d}}{4d}.$$

Accordingly, the change in the market supply is

$$\begin{array}{c} \begin{array}{cc} \Delta Q_{3}^{*}= & \left(-\frac{C+b-\sqrt{{\left(C+b\right)}^{2}+8d(N+1)-8d}}{4d})-(-\frac{C-\sqrt{C^{2}+\left(8N-8\right)d}}{4d}\right)\\ = & \frac{-b+\sqrt{C^{2}+2Cb+8dN+b^{2}}-\sqrt{C^{2}+(8N-8)d}}{4d}. \end{array} \end{array}$$

Further, we have

$${\left(\sqrt{C^{2}+2Cb+8dN+b^{2}}\right)}^{2}-{\left(\sqrt{C^{2}+(8N-8)d}\right)}^{2}=2Cb+b^{2}+8d.$$

If *d* > 0, then 2*Cb* + *b*^2^ + 8*d* > *b*^2^ > 0, and thus we have $\Delta Q_{3}^{*}>0$. Therefore, we can see that the entrant increases the market supply if there are diseconomies of scale, which decreases the product price since *P*(*Q*) = 1/*Q*. This aligns with the case of linear demand functions.

Next, we assume that *d* < 0. If 2*Cb* < −8*d*, then we have 2*Cb* + *b*^2^ + 8*d* < *b*^2^, which implies $\Delta Q_{3}^{*}>0$. Whereas, if 2*Cb* > −8*d*, then it follows that $\Delta Q_{3}^{*}<0$. This means that for the equilibrium $Q_{3}^{*}$, if economies of scale are small (|*d*| is particularly small), the new entrant leads to a decrease in the market supply. That is, when the economies of scale are limited, then the scale effect would dominate the competition effect by the new entrant and limit the market supply. The other positive equilibrium (i.e., the second one) satisfies

$$\Delta Q_{2}^{*}=\frac{-b-\sqrt{C^{2}+2Cb+8dN+b^{2}}+\sqrt{C^{2}+(8N-8)d}}{4d}.$$

When considering economies of scale, $\Delta Q_{2}^{*}>0$ holds if the scale effect is limited, which means that the new entrant increases the market supply and decreases the market price. Therefore, under economies of scale, $Q_{2}^{*}$ and $Q_{3}^{*}$ show opposite trends when new firms enter the market. That is, under economies of scale, the multiplicity of equilibria may alter the competition effects of firms’ entry, i.e., more firms may increase (decrease) the market competition with higher (lower) market supply, which depends on the selection of the equilibrium.

Regarding the effects of varying *C*, no matter *d* > 0 or *d* < 0, we have

$$\frac{\partial Q_{3}^{*}}{\partial C}=-\frac{1-\frac{C}{\sqrt{C^{2}+8dN-8d}}}{4d}<0.$$

If the number of firms is fixed, an increase in the aggregate initial marginal cost $C=\sum_{i=1}^{N}c_{i}$ will decrease the total market supply $Q_{3}^{*}$ and lead to a price increase. Furthermore, if *d* < 0, one can see that

$$\frac{\partial Q_{2}^{*}}{\partial C}=-\frac{1+\frac{C}{\sqrt{C^{2}+8dN-8d}}}{4d}>0.$$

Consequently, as the value of *C* varies, the two equilibria $Q_{2}^{*}$ and $Q_{3}^{*}$ may show opposite trends (i.e., *d* < 0). We can see that $Q_{3}^{*}$ maintains similar properties as in the standard Cournot competition, i.e., high costs lower the market supply. However, when considering the economy of scale (*d* < 0), we may have another equilibrium, i.e., $Q_{2}^{*}$, which contrasts the standard model, as it may increase the market supply as the cost increases.

In addition, as $Q_{2}^{*}$ is not applicable when *d* > 0, so for *d* < 0, we have

$$\frac{\partial Q_{2}^{*}}{\partial d}=-\frac{8N-8}{8\sqrt{C^{2}+8dN-8d}\;d}+\frac{C+\sqrt{C^{2}+8dN-8d}}{4d^{2}}>0.$$

That is, under the economies of scale, $Q_{2}^{*}$ always increases as the scale effect gets smaller. Furthermore,

$$\begin{array}{c} \begin{array}{cc} \frac{\partial Q_{3}^{*}}{\partial d} & =\frac{8N-8}{8\sqrt{C^{2}+8dN-8d}\;d}+\frac{C-\sqrt{C^{2}+8dN-8d}}{4d^{2}}\\ & =\frac{\sqrt{C^{2}+8dN-8d}\;C-C^{2}-4d\left(N-1\right)}{4\sqrt{C^{2}+8dN-8d}\;d^{2}}, \end{array} \end{array}$$

which implies that, when *d* > 0, $\partial Q_{3}^{*}/\partial d<0$ holds; but when *d* < 0, the sign of $\partial Q_{3}^{*}/\partial d$ can change depending on the distribution of other parameters. It means that, in the diseconomy of scale case, firms tend to further lower their output when such scale effect is stronger to achieve a more efficient level, and such incentive dominates other market factors. On the other hand, under the economies of scale, market output is undetermined because other market factors, such as market competition, production cost, etc, would also affect market output.

In sum, when there are two economically applicable (positive) equilibria, these two equilibria often display distinct patterns as the key parameter varies. Therefore, it is crucial to investigate whether these two equilibria would be achieved at the same time (with local stability). If this is the case, the economic system would exhibit much more complex behavior than a linear system. The difference in the initial state may result in the economic system converging to equilibrium through completely different patterns.

Then, we analyze the local stability of the equilibrium, and it is straightforward to show that an equilibrium Q* is locally stable in the one-dimensional map (2). If

$$|\frac{dQ(t+1)}{dQ(t)}{|}_{Q\left(t\right)=Q^{*}}|<1,$$

then Q* is locally stable. Otherwise, if

$$|\frac{dQ(t+1)}{dQ(t)}{|}_{Q\left(t\right)=Q^{*}}|>1,$$

then Q* is unstable.

Eq (3) indicates that the dynamics of *q*_*j*_ are equivalent to those of *Q*. Thus, the local stability of the original *N*-dimensional map (1) is equivalent to that of the aggregated one-dimensional one (2). Moreover, suppose we observe periodic or chaotic dynamics in market output. In that case, there are also periodic or chaotic patterns in the output of each firm in the market. This finding would considerably facilitate our empirical study, so that the macro-level business cycle may be built on similar micro-level patterns, and we have observed many firms have such experiences in reality.

One can see that

$$\frac{dQ(t+1)}{dQ(t)}{|}_{Q\left(t\right)=Q^{*}}=\frac{-d(N+1)Q^{*2}-2CQ^{*}+N+1}{2{\left(d\;Q^{*2}+1\right)}^{2}}.$$

Thus, the conditions of the local stability of the equilibrium include

$$\begin{array}{c} \text{Condition}\;\text{1}:\;\frac{dQ(t+1)}{dQ(t)}{|}_{Q\left(t\right)=Q^{*}}=\frac{-d(N+1)Q^{*2}-2CQ^{*}+N+1}{2{\left(d\;Q^{*2}+1\right)}^{2}}<1;\\ \text{Condition}\;\text{2}:\;\frac{dQ(t+1)}{dQ(t)}{|}_{Q\left(t\right)=Q^{*}}=\frac{-d(N+1)Q^{*2}-2CQ^{*}+N+1}{2{\left(d\;Q^{*2}+1\right)}^{2}}>-1. \end{array}$$

For the zero equilibrium $Q_{1}^{*}=0$, it is evident that

$$\frac{dQ(t+1)}{dQ(t)}{|}_{Q\left(t\right)=Q_{1}^{*}}=\frac{N+1}{2}>1,$$

which implies that $Q_{1}^{*}$ is unstable. Also, under the diseconomies of scale (*d* > 0), the second equilibrium satisfies that $Q_{2}^{*}<0$, and thus is not economically applicable.

Next, we only focus on the stability analysis of $Q_{3}^{*}$. Except for the boundary (*d* = *C*^2^(*N* − 5)/32), the following theorem provides a necessary and sufficient condition for the local stability of the unique Cournot-Nash equilibrium $Q_{3}^{*}$.

**Theorem 2**. *Suppose we have diseconomies of scale, i.e., d* > 0. *If*

$$d>\frac{C^{2}\left(N-5\right)}{32},$$

*then* $Q_{3}^{*}$ *is locally stable. But, if*

$$d<\frac{C^{2}\left(N-5\right)}{32},$$

*then* $Q_{3}^{*}$ *is locally unstable*.

*Proof*. Regarding Condition 1, we can plug in the closed form of $Q_{3}^{*}$ and obtain the equivalent condition

$$\frac{-2(C^{2}+(6N-2)d)C\sqrt{(8N-8)d+C^{2}}+2(C^{2}+8d(N-1))(C^{2}+2d(N+1))}{{\left(-C\sqrt{(8N-8)d+C^{2}}+C^{2}+4d(N+1)\right)}^{2}}>0.$$

Since the denominator is positive, the above inequality is equivalent to

$$\begin{array}{c} \begin{array}{cc} & {\left(2(C^{2}+2d(N+1))(C^{2}+8d(N-1))\right)}^{2}\\ & -{\left(2C(C^{2}+(6N-2)d)\sqrt{(8N-8)d+C^{2}}\right)}^{2}>0. \end{array} \end{array}$$

Accordingly, we have

$$128({\left(N+1\right)}^{2}d+C^{2})((8N-8)d+C^{2})d^{2}(N-1)>0,$$

which always holds if *d* > 0.

Regarding Condition 2, the equivalent condition after plugging in the equation of $Q_{3}^{*}$ is

$$\frac{-2(C^{2}+2d(N+5))C\sqrt{(8N-8)d+C^{2}}+2C^{4}+12d(N+1)C^{2}+64d^{2}(N+1)}{{\left(-C\sqrt{(8N-8)d+C^{2}}+C^{2}+4d(N+1)\right)}^{2}}>0.$$

Since the denominator is positive, the above inequality can be transformed into

$$\begin{array}{c} \begin{array}{cc} & {\left(2C^{4}+12d(N+1)C^{2}+64d^{2}(N+1)\right)}^{2}\\ & -{\left(2C(C^{2}+2d(N+5))\sqrt{(8N-8)d+C^{2}}\right)}^{2}>0. \end{array} \end{array}$$

Further simplification gives

$$128({\left(N+1\right)}^{2}d+C^{2})d^{2}(-32d+C^{2}(N-5))<0.$$

Since *d* > 0, the above inequality is equivalent to *d* > *C*^2^(*N* − 5)/32, which completes our proof.

According to Theorem 2, the stability of the equilibrium $Q_{3}^{*}$ is guaranteed if the number of firms is less than or equal to five under diseconomies of scale. In the real world, the number of firms is often small in highly disaggregated markets. In this regard, if we apply our model to analyze real economic problems, a unique equilibrium exists, which is always stable. In this case, complicated dynamics such as periodic solutions and chaos in the economic system should not occur. Even if the number of firms is large, Theorem 2 shows that the stability of the equilibrium is guaranteed as long as diseconomies of scale are sufficiently high, i.e., *d* is large enough.

Similar to the findings of Fisher [4], we observe some interesting features of our model. Under diseconomies of scale, Theorem 2 reveals that an upshift in the firm’s initial marginal cost $C_{i}^{\prime }\left(0\right)=c_{i}$ will lead to an increase in *C*, which may destabilize our model. However, the faster the firms’ marginal cost increases (a larger *d*), the more stable our model would be. Moreover, the more firms engage in our model, *C* and *N* increase simultaneously, making our model unstable.

Under economies of scale (*d* < 0), the stability analysis is quite complicated if the number of firms *N* is unknown. Below, we only discuss the duopoly (*N* = 2) and triopoly (*N* = 3) markets. When we focus on some narrowly defined industries, the oligopoly market structure, with two or three large companies, is quite common, such as telecommunication, oil, and natural gas sectors, to name a few.

**Proposition 2**. *Suppose we have economies of scale, i.e., d* < 0, *and consider a duopoly market* (*N* = 2). *If C*^2^ > −32*d*/3, *then the second equilibrium satisfies* $Q_{2}^{*}>0$ *and is locally stable. If* *C*^2^ > −8*d*, *then the third equilibrium satisfies* $Q_{3}^{*}>0$ *and is locally stable*.

*Proof*. By plugging in the equation of $Q_{3}^{*}$, we transform Condition 1 into

$$\begin{array}{c} \frac{2C^{4}-2C^{3}\sqrt{C^{2}+8d}+28C^{2}d-20C\sqrt{C^{2}+8d}\;d+96d^{2}}{{\left(C^{2}-C\sqrt{C^{2}+8d}+12d\right)}^{2}}>0. \end{array}$$ (8)

Since *d* < 0, we have $\sqrt{C^{2}+8d}<C$, which implies $2C^{4}-2C^{3}\sqrt{C^{2}+8d}+96d^{2}>0$ and $28C^{2}d-20C\sqrt{C^{2}+8d}\;d<0$. Accordingly, we should consider the sign of

$$\begin{array}{c} {\left(2C^{4}-2C^{3}\sqrt{C^{2}+8d}+96d^{2}\right)}^{2}-{\left(28C^{2}d-20C\sqrt{C^{2}+8d}\;d\right)}^{2}\\ =-32d(C^{6}-C^{5}\sqrt{C^{2}+8d}-100C^{2}d^{2}+120C\sqrt{C^{2}+8d}\;d^{2}-288d^{3}). \end{array}$$

It can be shown that the above expression is positive. Therefore, the inequality (8) is guaranteed, which means that Conditions 1 always holds.

We substitute the equation of $Q_{3}^{*}$ into Condition 2 and obtain that

$$\frac{2C^{4}-2C^{3}\sqrt{C^{2}+8d}+36C^{2}d-28C\sqrt{C^{2}+8d}\;d+192d^{2}}{{\left(C^{2}-C\sqrt{C^{2}+8d}+12d\right)}^{2}}>0.$$

Similar to the above discussions, this inequality holds. In short, $Q_{3}^{*}$ is always locally stable.

Regarding $Q_{2}^{*}$, we plug its equation into Condition 1 and acquire that

$$\frac{2C^{4}+2C^{3}\sqrt{C^{2}+8d}+28C^{2}d+20C\sqrt{C^{2}+8d}\;d+96d^{2}}{{\left(C^{2}+C\sqrt{C^{2}+8d}+12d\right)}^{2}}>0.$$

If the condition for $Q_{2}^{*}$’s existence, i.e., *C*^2^ + 8*d* > 0, is satisfied, the above inequality is equivalent to *C*^2^ > −9*d*.

By substituting the equation of $Q_{2}^{*}$, we know that Condition 2 is equivalent to

$$\frac{2C^{4}+2C^{3}\sqrt{C^{2}+8d}+36C^{2}d+28C\sqrt{C^{2}+8d}\;d+192d^{2}}{{\left(C^{2}+C\sqrt{C^{2}+8d}+12d\right)}^{2}}>0.$$

Again, if the condition for $Q_{2}^{*}$’s existence holds, then the above inequality is equivalent to *C*^2^ < −9*d* or *C*^2^ > −32*d*/3. In sum, Conditions 1 and 2 are satisfied at $Q_{2}^{*}$ if and only if *C*^2^ > −32*d*/3. The proof is completed.

**Proposition 3**. *Suppose we have economies of scale, i.e., d* < 0, *with a triopoly market* (*N* = 3). *If C*^2^ > −16*d*, *then we have* $Q_{2}^{*}>0$ *and* $Q_{3}^{*}>0$, *of which both are locally stable*.

*Proof*. Regarding the stability of $Q_{2}^{*}$, Conditions 1 and 2 can be simplified as:

$$\frac{2(C^{2}+16d)(C^{2}+C\sqrt{C^{2}+16d}+8d)}{{\left(C^{2}+C\sqrt{C^{2}+16d}+16d\right)}^{2}}>0.$$

If the condition for $Q_{2}^{*}$’s existence holds, i.e., *C*^2^ + 16*d* > 0 or *C*^2^ > −16*d*, then it implies that $C^{2}+C\sqrt{C^{2}+16d}+8d>0$. Therefore, we get the above inequality.

Regarding the stability of $Q_{3}^{*}$, Conditions 1 and 2 can be transformed into

$$\frac{2(C^{2}+16d)(C^{2}-C\sqrt{C^{2}+16d}+8d)}{{\left(C^{2}-C\sqrt{C^{2}+16d}+16d\right)}^{2}}>0.$$

Also, if *C*^2^ + 16*d* > 0 or *C*^2^ > −16*d*, then we get $C^{2}-C\sqrt{C^{2}+16d}+8d>0$, which implies the above inequality. The proof is completed.

Both the above propositions reveal that the model may have two stable and positive equilibria under economies of scale. Multiple equilibria bring challenges to comparative statics analysis and reduce the predictive power of our model. Particularly, the conditions for multiple stable equilibria cannot show us which equilibrium should be selected. In the model’s dynamics, the final state where the model converges depends on the initial state (*q*_1_(0), *q*_2_(0), …, *q*_*N*_(0)), i.e., each firm’s output at *t* = 0. Choosing the equilibrium requires a global analysis of the dynamic properties of our model, which is beyond the scope of this paper, and we leave it for future research.

Finally, we consider the special case of constant marginal costs, i.e., *d*_1_ = *d*_2_ = ⋯ = *d*_*N*_ = 0. In this case, the aggregation map (2) becomes

$$\begin{array}{c} Q\left(t+1\right)=\frac{1}{2}(Q\left(t\right)+NQ(t)-CQ^{2}\left(t\right)). \end{array}$$ (9)

We can see that the above system has two equilibria, i.e., 0 and (*N* − 1)/*C*. Also, if the number of firms is fixed, an increase in the marginal cost *c*_*i*_ will increase *C* and thus decrease the market supply (*N* − 1)/*C*. In addition, we also need to consider the impact of new entrants into the market. Assuming that the marginal cost of the new entrant is *b* > 0 and the number of firms then becomes *N* + 1, the variation of the market supply would be

$$\frac{N}{C+b}-\frac{N-1}{C}=\frac{C-(N-1)b}{(C+b)C}.$$

Thus, if the marginal cost of the new entrant satisfies that *b* < *C*/(*N* − 1), the market supply will grow, and vice versa. This aligns with the standard Cournot model with constant marginal cost settings, as more/less efficient firms enter the market, which decreases/increases the market average cost level, then the total supply will increase/decrease.

Regarding the stability when *d*_*i*_ = 0, Bischi et al. [19] provide comprehensive results. As a comparison, we restate the facts found by Bischi et al. [19] below. The main idea of their approach is transforming map (9) into the Logistic mapping of the form *x*(*t* + 1) = *ux*(*t*)(1 − *x*(*t*)). Since the Logistic mapping has been well studied in the literature, we can get the dynamic behaviors of our model with *d*_*i*_ = 0:

- if *N* ≤ 3, then map (9) converges monotonically to the positive equilibrium (*N* − 1)/*C*;
- if 4 ≤ *N* ≤ 5, then map (9) converges oscillatingly to the positive equilibrium (*N* − 1)/*C*;
- if *N* = 6, then map (9) displays a stable periodic orbit with order 4;
- if *N* > 6, then map (9) exhibits complex dynamics such as chaos.

Therefore, in the special case of constant marginal costs, when more firms engage in market competition, the market dynamics get complex, which may be due to more complicated interactions and strategic dependence among market participants.

## 4 Heterogeneous *d*_*i*_

The case of heterogeneous *d*_*i*_ is much more complex, and this section investigates the duopoly game with heterogeneous *d*_*i*_. The iteration map of the duopoly can be written as

$$\begin{array}{c} \left\{ \begin{array}{cc} q_{1}\left(t+1\right)= & \;\frac{2\;q_{1}\left(t\right)+q_{2}\left(t\right)-c_{1}{\left(q_{1}\left(t\right)+q_{2}\left(t\right)\right)}^{2}}{2(d_{1}{\left(q_{1}\left(t\right)+q_{2}\left(t\right)\right)}^{2}+1)},\\ q_{2}\left(t+1\right)= & \;\frac{2\;q_{2}\left(t\right)+q_{1}\left(t\right)-c_{2}{\left(q_{1}\left(t\right)+q_{2}\left(t\right)\right)}^{2}}{2(d_{2}{\left(q_{1}\left(t\right)+q_{2}\left(t\right)\right)}^{2}+1)}. \end{array}\right. \end{array}$$ (10)

From an economic point of view, it is important to identify the number of positive equilibria, namely $\left(q_{1}^{*},q_{2}^{*}\right)$ such that $q_{1}^{*}>0,q_{2}^{*}>0$. Accordingly, one can obtain the following polynomial system of equilibrium.

$$\begin{array}{c} \left\{ \begin{array}{cc} & 2\;q_{1}^{*}+q_{2}^{*}-c_{1}{\left(q_{1}^{*}+q_{2}^{*}\right)}^{2}-2(d_{1}{\left(q_{1}^{*}+q_{2}^{*}\right)}^{2}+1)q_{1}^{*}=0,\\ & 2\;q_{2}^{*}+q_{1}^{*}-c_{2}{\left(q_{1}^{*}+q_{2}^{*}\right)}^{2}-2(d_{2}{\left(q_{1}^{*}+q_{2}^{*}\right)}^{2}+1)q_{2}^{*}=0,\\ & q_{1}^{*}>0,\;q_{2}^{*}>0. \end{array}\right. \end{array}$$ (11)

The analytical solution of the system (11) is quite complicated if *d*_1_ ≠ *d*_2_. Therefore, it is impossible to analyze the equilibrium’s local stability and bifurcations. Instead, we employ a more general approach to overcome the challenge that closed-form equilibria cannot be obtained explicitly. Our approach is based on symbolic computation, such as the triangular decomposition method.

The triangular decomposition method, which is an extension of the Gaussian elimination method, allows us to analyze the equilibria of non-linear economic models. The triangular decomposition and Gaussian elimination can transform a system into triangular forms. However, the triangular decomposition method is feasible for polynomial systems, while the Gaussian elimination method is just for linear systems. Readers can refer to [24, 36–39] for more information on triangular decomposition. Using the triangular decomposition method, we can decompose the solutions of system (11) into the zeros of two polynomial sets: $T_{1}=[q_{2}^{*},\;q_{1}^{*}]$ and $T_{2}=[T_{2},T_{1}]$, where *T*_2_ and *T*_1_ can be found in S1 Appendix.

The zero of $T_{1}$ corresponds to the origin (0, 0). Moreover, the non-vanishing equilibria can be computed from $T_{2}$. The polynomial *T*_1_ in $T_{2}$ is univariate in $q_{1}^{*}$ with degree 4. Furthermore, the polynomial *T*_2_ in $T_{2}$ is linear in $q_{2}^{*}$. Consequently, after solving $q_{1}^{*}$ from *T*_1_, we can substitute $q_{1}^{*}$ into *T*_2_ and get $q_{2}^{*}$. As *T*_1_ is 4-th degree in $q_{1}^{*}$, we know there are at most 4 real solutions. Thus, their analytical expressions exist but are quite complicated, though.

For simplicity, we suppose that *c*_1_ = *c*_2_ = 1 hereafter. In this case, the analytical solutions of $T_{2}$ are still complicated. Thus, it is still challenging to identify the number of positive equilibria (i.e., the real solution of system (11)). However, Li and Wang [25] propose an algebraic algorithm to systematically determine the number of the equilibria satisfying certain inequalities without obtaining the closed-form expressions. Readers can refer to Section 3 of [25] for additional information on their algorithm. The main idea of their algorithm is to compute the so-called border polynomial, whose zeros divide the parameter space into several regions such that the number of real solutions satisfying the inequalities is invariant within each region. Consequently, we need to select at least one sample point from each region and identify the solution number at the selected sample point.

In practice, the selection of sample points could be computationally burdensome in general but could be automated using, e.g., the partial cylindrical algebraic decomposition (PCAD) method [40]. We leverage the **SamplePoints** function in the Maple package **RegularChains** to select sample points. For system (11), we obtain the border polynomial *BP*_1_, which can be found in the S1 Appendix. Accordingly, the PCAD method generates 98 sample points of the parameter plane {(*d*_1_, *d*_2_)| − ∞ < *d*_1_ < + ∞, −∞ < *d*_2_ < + ∞} which is divided by *BP*_1_ = 0. All the selected sample points of (*d*_1_, *d*_2_) and their corresponding number of positive equilibria are listed as follows:

- zero positive equilibria:
  $$\begin{array}{c} \begin{array}{cc} & (-1,-2),\;(-1,-3/4),\;(-1,1/8),\;(-1,1),\;(-19/32,-1),\;(-19/32,-9/16),\\ & (-19/32,1/128),\;(-37/64,-1),\;(-37/64,-9/16),\;(-71/128,-1),\;(-71/128,-17/32),\\ & (-17/32,-1),\;(-17/32,-33/64),\;(1/16,-2),\;(1/16,-1),\;(3/32,-2),\;(3/32,-1),\\ & (1/8,-2),\;(1/8,-1),\;(1/8,-5/8),\;(1/2,-14),\;(1/2,-3),\;(1/2,-1),\;(5/4,-123),\;\\ & (5/4,-4),\;(5/4,-1),\;(2,-424),\;(2,-5),\;(2,-2),\;(2,-3/4); \end{array} \end{array}$$
- one positive equilibrium:
  $$\begin{array}{c} \begin{array}{cc} & (-1,-1/4),\;(-19/32,-1/4),\;(-37/64,-1/4),\;(-71/128,-3/8),\;(-17/32,-7/16),\;\\ & (-1/4,-2),\;(-1/4,-3/4),\;(-1/4,1/32),\;(-1/4,61/1024),\;(-1/4,1),\;(-1/8,-2),\;\\ & (-1/8,-1),\;(-1/8,1/32),\;(-1/8,19/512),\;(-1/8,1),\;(1/16,-3/8),\;(1/16,-1/8),\;\\ & (1/16,-1/64),\;(1/16,1/32),\;(1/16,1),\;(3/32,-1/4),\;(3/32,-1/32),\;(3/32,1/16),\;\\ & (3/32,1),\;(1/8,-1/4),\;(1/8,-1/32),\;(1/8,1/16),\;(1/8,1),\;(1/2,-1/4),\;(1/2,-1/8),\;\\ & (1/2,1/4),\;(1/2,1),\;(5/4,-1/4),\;(5/4,1),\;(5/4,2),\;(2,-1/4),\;(2,1),\;(2,3); \end{array} \end{array}$$
- two positive equilibria:
  $$\begin{array}{c} \begin{array}{cc} & (-1,13),\;(-19/32,1/16),\;(-19/32,1),\;(-37/64,1/16),\;(-37/64,1),\;(-71/128,1/16),\;\\ & (-71/128,1),\;(-17/32,1/16),\;(-17/32,1),\;(-1/4,-3/8),\;(-1/4,-1/8),\;(-1/8,-1/4),\;\\ & (-1/8,-1/16),\;(1/16,-9/16),\;(3/32,-9/16),\;(3/32,-65/128),\;(1/8,-9/16),\;\\ & (1/2,-5/8),\;(5/4,-21/32),\;(5/4,-5/8),\;(2,-5/8); \end{array} \end{array}$$
- three positive equilibria:
  $$\begin{array}{c} \begin{array}{cc} & (-37/64,-1/16),\;(-71/128,-291/1024),\;(-71/128,-1/8),\;(-71/128,-1/16),\;\\ & (-17/32,-51/128),\;(-17/32,-1/4),\;(-17/32,-1/32),\;(-1/4,-17/32),\;(-1/8,-9/16). \end{array} \end{array}$$

**Fig 1:**
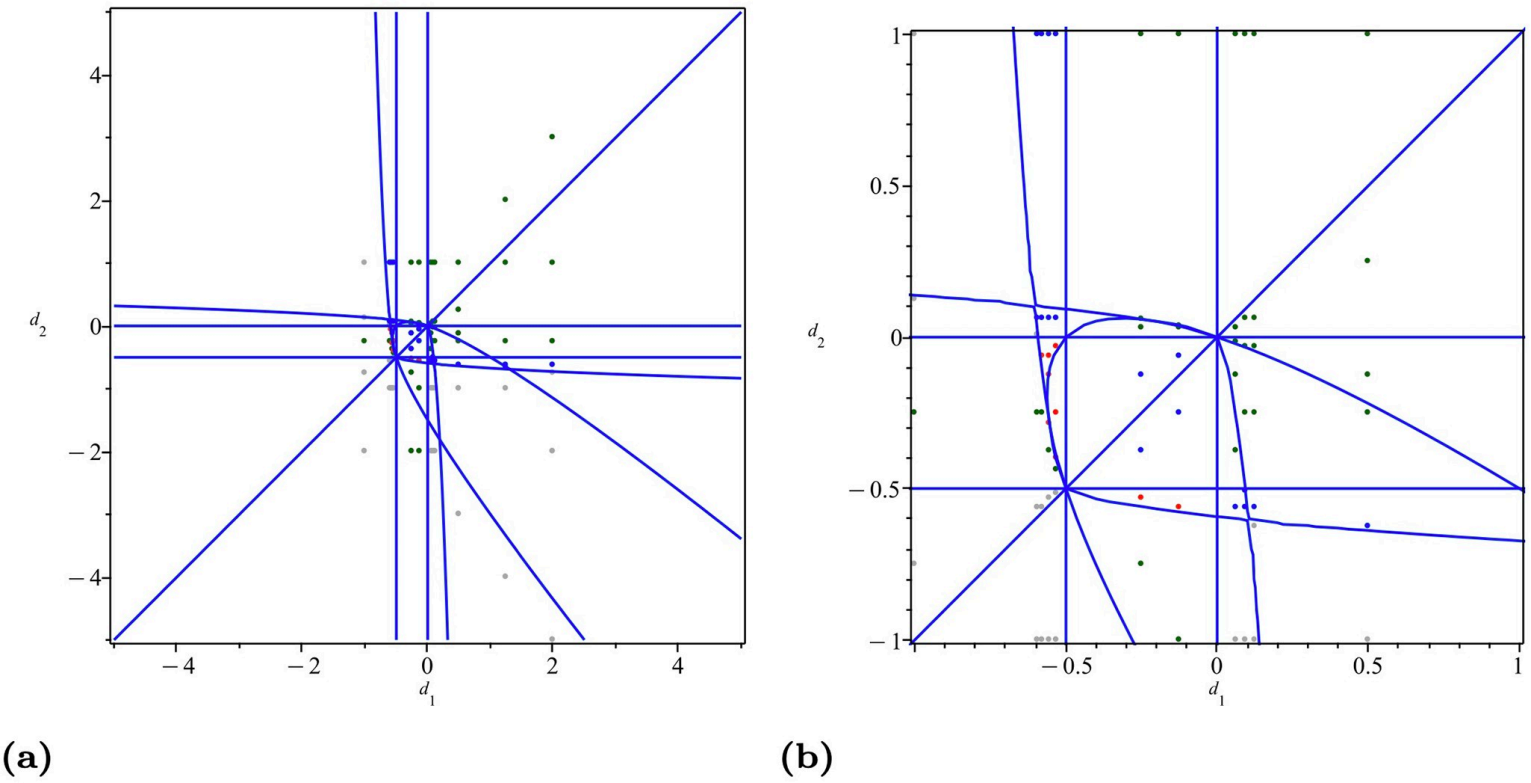
The curve of *BP*_1_ = 0 in the parameter plane. The selected sample points corresponding to zero, one, two, and three positive equilibria are marked in gray, green, blue, and red, respectively. (a) (*d*_1_, *d*_2_) ∈ (−5, 5) × (−5, 5). (b) (*d*_1_, *d*_2_) ∈ (−1, 1) × (−1, 1).

In Fig 1, we demonstrate the parameter plane. The blue curve is the zeros of the border polynomial *BP*_1_. We color the selected sample points corresponding to zero, one, two, and three positive equilibria in gray, green, blue, and red, respectively. The enlarged view is given in Fig 1(b), where additional details around the origin can be found.

To investigate the local stability of the equilibrium, the following Jacobian matrix plays a crucial role.

$$J=[\begin{array}{cc} J_{11} & J_{12}\\ J_{21} & J_{22} \end{array}]=[\begin{array}{cc} \frac{-q_{1}^{*}\left(q_{1}^{*}+q_{2}^{*}\right)d_{1}-c_{1}q_{1}^{*}-c_{1}q_{2}^{*}+1}{{\left(d_{1}{\left(q_{1}^{*}+q_{2}^{*}\right)}^{2}+1\right)}^{2}} & \frac{\left(-3q_{1}^{*2}-4q_{1}^{*}q_{2}^{*}-q_{2}^{*2}\right)d_{1}-2c_{1}q_{1}^{*}-2c_{1}q_{2}^{*}+1}{2{\left(d_{1}{\left(q_{1}^{*}+q_{2}^{*}\right)}^{2}+1\right)}^{2}}\\ \frac{-\left(q_{1}^{*}+3q_{2}^{*}\right)\left(q_{1}^{*}+q_{2}^{*}\right)d_{2}-2c_{2}q_{1}^{*}-2c_{2}q_{2}^{*}+1}{2{\left(d_{2}{\left(q_{1}^{*}+q_{2}^{*}\right)}^{2}+1\right)}^{2}} & \frac{-q_{2}^{*}\left(q_{1}^{*}+q_{2}^{*}\right)d_{2}-c_{2}q_{1}^{*}-c_{2}q_{2}^{*}+1}{{\left(d_{2}{\left(q_{1}^{*}+q_{2}^{*}\right)}^{2}+1\right)}^{2}} \end{array}].$$

In particular, at the zero equilibrium *E*_0_ = (0, 0), the corresponding Jacobian matrix is

$$\begin{array}{c} J\left(E_{0}\right)=[\begin{array}{cc} 1 & 1/2\\ 1/2 & 1 \end{array}]. \end{array}$$

Thus, its eigenvalues are 1/2 and 3/2, which implies that the zero equilibrium *E*_0_ is locally unstable.

In general, the Jury criterion can be employed to determine the local stability of multi-dimensional discrete dynamic systems. According to the Jury criterion, an equilibrium $\left(q_{1}^{*},q_{2}^{*}\right)$ of map (10) is locally stable if the following three conditions are fulfilled:

1.
$CD_{1}^{J}\equiv 1-\text{Tr}(J\left(\left(q_{1}^{*},q_{2}^{*}\right)\right)+\text{Det}\left(J\left(q_{1}^{*},q_{2}^{*}\right)\right)>0$ ,
2.
$CD_{2}^{J}\equiv 1+\text{Tr}\left(J\left(q_{1}^{*},q_{2}^{*}\right)\right)+\text{Det}\left(J\left(q_{1}^{*},q_{2}^{*}\right)\right)>0$ ,
3.
$CD_{3}^{J}\equiv 1-\text{Det}\left(J\left(q_{1}^{*},q_{2}^{*}\right)\right)>0$ ,

where Tr(*J*) = *J*_11_ + *J*_22_ and Det(*J*) = *J*_11_*J*_22_ − *J*_12_*J*_21_ are the trace and the determinant of *J*, respectively.

**Fig 2:**
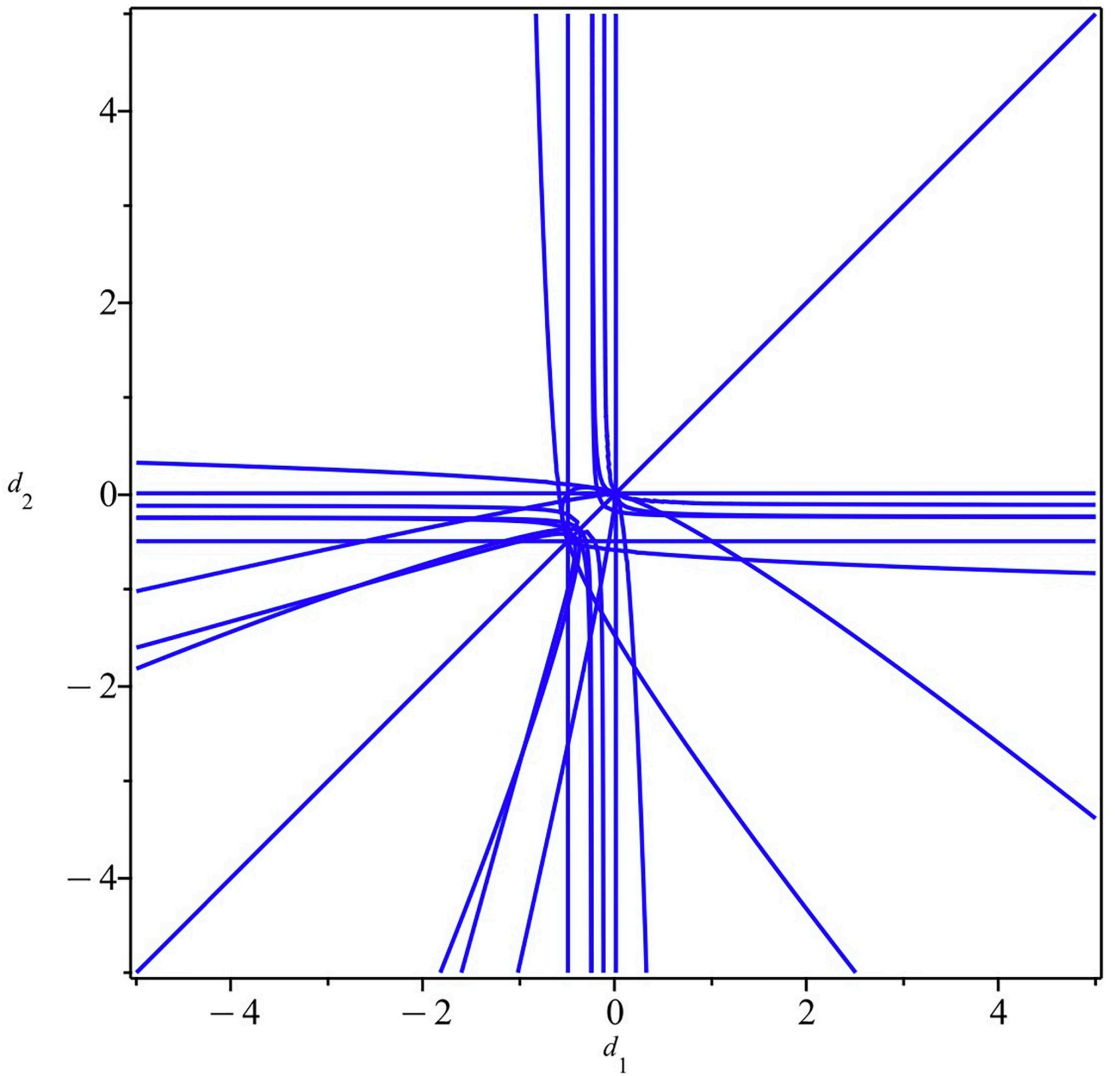
The curve of *BP*_2_ = 0 in the parameter plane.

Therefore, if we focus on the stable positive equilibria of map (10), we need to consider system (11) together with the inequalities $CD_{1}^{J}>0$, $CD_{2}^{J}>0$ and $CD_{3}^{J}>0$. Similarly, we also compute the corresponding border polynomial *BP*_2_ (See the S1 Appendix). This border polynomial is more complex than *BP*_1_, the zeros plotted in Fig 2. Furthermore, for the parameter space divided by *BP*_2_ = 0, the PCAD method generates 1016 sample points of (*d*_1_, *d*_2_). We do not list all of them due to space limitations. Based on our computation, two stable positive equilibria, at most, exist at these sample points. That is, our duopoly model with heterogeneous *d*_*i*_ would have two stable and applicable equilibria, at most. Therefore, there can be zero, one, or two stable positive equilibria. We list a few selected sample points and their corresponding numbers of stable positive equilibria as follows:

- zero stable positive equilibria: (−3, −14), (−31/32, −3/4), (−31/32, 1/8);
- one stable positive equilibrium: (−31/32, −1/8), (−21/8, −1/8), (−21/8, 499);
- two stable positive equilibria: (−1/8, −9/16), (−71/128, −1/16), (−275/512, −1/32).

*Remark* 2. From an economic point of view, we are interested in cases with multiple stable and applicable equilibria. Then, the equilibrium selection issue arises. According to the results above, we know that at least at three points of (*d*_1_, *d*_2_) (i.e., (−1/8, −9/16), (−71/128, −1/16), and (−275/512, −1/32)) there are two stable positive equilibria. In the next section, we perform numerical simulations to explore the delimitation of the boundaries of their basins of attraction.

Moreover, we derive the following proposition according to the results above.

**Proposition 4**. *Let c*_1_ = *c*_2_ = 1. *The duopoly game with heterogeneous d*_*i*_ *always has one and only one stable positive equilibrium if d*_1_ > 0 *and d*_2_ > 0.

*Proof*. All the selected sample points in the first quadrant {(*d*_1_, *d*_2_)|*d*_1_ > 0, *d*_2_ > 0} include:

$$\begin{array}{c} \begin{array}{cc} & (1/64,1/128),\;(1/64,1),\;(1/32,1/64),\;(1/32,1),\;(1/16,1/32),\;(1/16,1),\;\\ & (3/32,1/16),\;(3/32,1),\;(1/8,1/16),\;(1/8,1),\;(3/16,1/8),\;(3/16,1),\;(1/4,1/8),\;\\ & (1/4,1),\;(7/16,1/4),\;(7/16,1),\;(1/2,1/4),\;(1/2,1),\;(3/4,1/2),\;(3/4,1),\;\\ & (5/4,1),\;(5/4,2),\;(2,1),\;(2,3). \end{array} \end{array}$$

It can be verified that the system (11) combined with the inequalities $CD_{1}^{J}>0$, $CD_{2}^{J}>0$ and $CD_{3}^{J}>0$ has one and only one real solution at each of the above points. This implies that the duopoly game of heterogeneous *d*_*i*_ always has one and only one stable positive equilibrium if *d*_1_ > 0 and *d*_2_ > 0.

In sum, Proposition 4 reveals that for the duopoly game with heterogeneous *d*_*i*_, if we have economies of scale, there is one and only one applicable equilibrium, which is similar to the case of homogeneous *d*_*i*_. On the other hand, for the duopoly game with heterogeneous *d*_*i*_, if we have diseconomies of scale, multiple stable equilibria may exist depending on the values of *d*_1_ and *d*_2_.

## 5 Numerical simulations

In this section, we conduct numerical simulations to explore the complex dynamics of our model. First, assume that *d*_*i*_ is identical and consider the aggregate map (2). Recall that Eq (3) reveals that the dynamics of *q*_*j*_ correspond to those of *Q*. Thus, if we find periodic or chaotic dynamics in numerical simulations on market output, we can conclude that there are corresponding periodic or chaotic patterns in each firm’s output involved in the Cournot competition. That is, if we have homogeneous *d*_*i*_, we need to focus on the exploration of the aggregate map (2).

**Fig 3:**
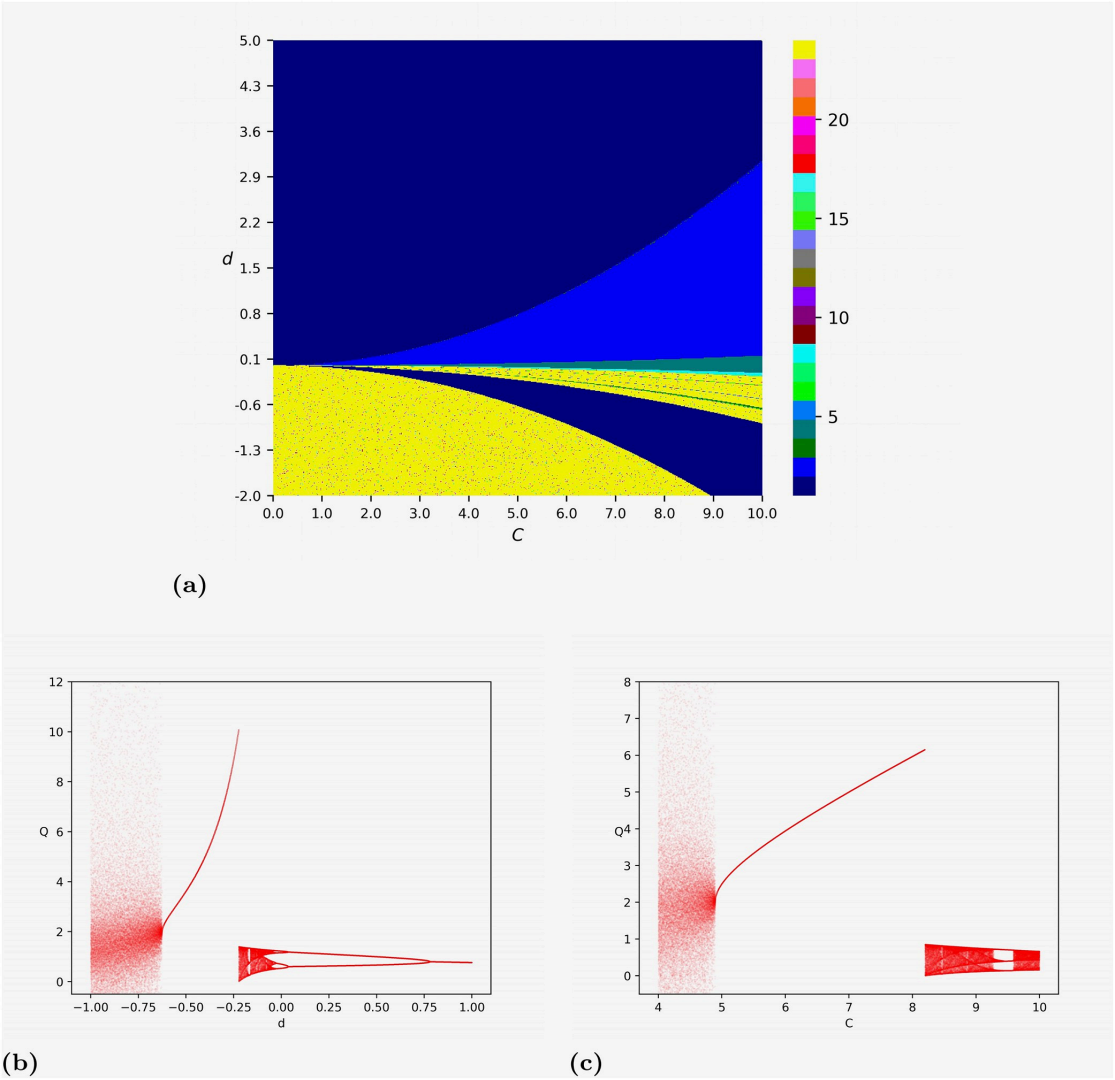
Numerical simulations on map (2) with *N* = 6. The initial state of the iterations is set to be (*q*_1_(0), *q*_2_(0)) = (0.1, 0.1). (a) The two-dimensional bifurcation diagram by varying (*C*, *d*) ∈ (0, 10) × (−2, 5). (b) The one-dimensional bifurcation diagram by fixing *C* = 5 and varying *d* ∈ (−1, 1). (c)The one-dimensional bifurcation diagram by fixing *d* = −0.6 and varying *C* ∈ (4, 10).

According to Theorem 2, we know that the equilibrium $Q_{3}^{*}$ loses its local stability if *d* < *C*^2^(*N* − 5)/32 under decreasing returns to scale. This would be the case if *N* ≥ 6. For example, Fig 3 shows the bifurcation diagrams if six firms are involved in the Cournot competition, i.e., *N* = 6.

Fig 3(b) shows the dynamic transitions of the Cournot game with six firms by fixing *C* = 5 and varying the parameter *d*. From Fig 3(b), we can see that the trajectory approaches the equilibrium $Q_{3}^{*}$ if *d* > 0.790895448. In particular, when *d* = 1, we have $Q_{3}^{*}=0.7655644370$. The equilibrium bifurcates into a 2-cycle orbit when *d* = 0.790895448, then transitions to a 4-cycle orbit when *d* = 0.036518259. If the value of *d* further decreases, chaotic dynamics occur through a cascade of period-doubling bifurcations. When *d* = −0.223611806, the chaotic behaviors suddenly disappear, and the trajectory converges to the equilibrium $Q_{2}^{*}=10.06983388$, which is much larger than $Q_{3}^{*}$ when *d* > 0.790895448. If the value of *d* further decreases and equals −0.625812906, the equilibrium loses its stability through a border collision bifurcation, and chaos suddenly occurs with a huge range.

In Fig 3(c), we fix *d* = −0.6 and varying *C* from 4 to 10. We can see that the dynamics of the model are complicated if *d* < 4.900450225. However, when 4.900450225 < *d* < 8.19909955, the trajectory approaches the stable equilibrium $Q_{2}^{*}$. If the value of *d* increases, chaos with a smaller range occurs. Furthermore, we can see that there exists a 3-cycle orbit if 9.44172086 < *d* < 9.585792896. In short, the trajectory will converge to a stable state only if *C* takes moderate values. The chaotic dynamics reappear through a border collision if the value of *C* is small enough.

Furthermore, Fig 3(a) depicts the two-dimensional bifurcation diagram to show more complex dynamics concerning parameters *C* and *d*. We use different colors to mark parameter points corresponding to periodic orbits with different orders and use yellow points to represent the parameter points where complex dynamics such as chaos occur, or the trajectory diverges (approaches ∞). We find that for different values of *C* (resp. *d*), there exist similar patterns as that discovered in Fig 3(b) (resp. Fig 3(c)). If we have economies of scale, i.e., *d* < 0, the model dynamics are complicated when the value of *C* is small or big enough. However, the dynamic behaviors of our model would be simpler under decreasing return to scale (*d* > 0) than increasing return to scale (*d* < 0).

**Fig 4:**
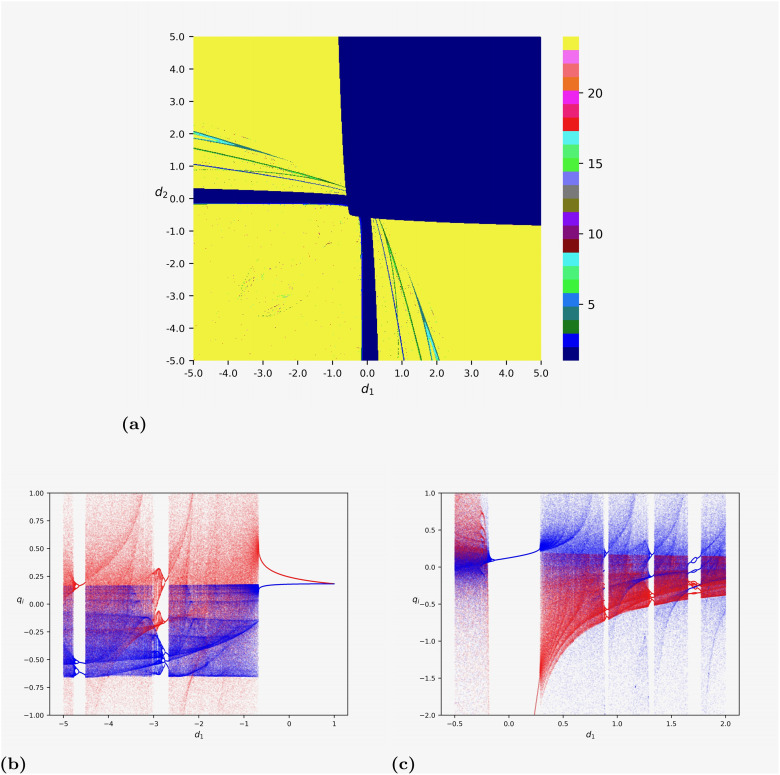
Numerical simulations on map (10) with *c*_1_ = *c*_2_ = 1. The initial state of the iterations is set to be (*q*_1_(0), *q*_2_(0)) = (0.1, 0.1). (a) The two-dimensional bifurcation diagram by varying (*d*_1_, *d*_2_) ∈ (−5, 5) × (−5, 5). (b) The one-dimensional bifurcation diagram by fixing *d*_2_ = 1 and varying *d*_1_ ∈ (−5, 1). The trajectories of *q*_1_ and *q*_2_ are marked in red and blue, respectively. (c) The one-dimensional bifurcation diagram by fixing *d*_2_ = −4 and varying *d*_1_ ∈ (−0.5, 2). The trajectories of *q*_1_ and *q*_2_ are marked in red and blue, respectively.

Then, we focus on the duopoly game of heterogeneous *d*_*i*_ considered above. Fig 4 reports the results of numerical simulations of the two-dimensional discrete dynamic map (10) with *c*_1_ = *c*_2_ = 1.

Similar to the discussion above, we set the initial state of our numerical simulations to be (*q*_1_(0), *q*_2_(0)) = (0.1, 0.1). In Fig 4(a), we plot the two-dimensional bifurcation diagram by varying (*d*_1_, *d*_2_) in the region (−5, 5) × (−5, 5). We can see that for all points in the first quadrant {(*d*_1_, *d*_2_)|*d*_1_ > 0, *d*_2_ > 0}, the trajectory always approaches some stable equilibrium, which is consistent with the result of Proposition 4. Furthermore, in the other three quadrants, some narrow regions around the axes *d*_1_ = 0 and *d*_2_ = 0, where the corresponding trajectories converge to certain stable states. Complex dynamics such as periodic orbits and chaos appear at most of the parameter points in the other three quadrants, meaning that the economic system may become complicated if, for some firms, the effect of increasing returns to scale is large enough.

Fig 4(b) depicts the one-dimensional bifurcation diagram by fixing *d*_2_ = 1 and varying *d*_1_ ∈ (−5, 1). The trajectories of *q*_1_ and *q*_2_ are marked in red and blue, respectively. We observe that there exists a stable equilibrium when *d*_1_ > −0.674837419. If *d*_1_ decreases and becomes smaller than −0.674837419, chaotic dynamics would occur through a border collision bifurcation, where the trajectory range of *q*_1_ is wider than that of *q*_2_. When *d*_1_ = −2.670835418, a 3-cycle orbit appears and loses its stability through a series of period-doubling bifurcations. After that, chaotic attractors reappear. When *d*_1_ = −4.513756878, a 2-cycle orbit occurs and bifurcates into chaos through a cascade of period-doubling bifurcations.

In Fig 4(c), we show the bifurcation diagram by fixing *d*_2_ = −4 and varying *d*_1_ ∈ (−0.5, 2). Same as Fig 4(b), we mark the trajectories of *q*_1_ and *q*_2_ in red and blue, respectively. We can see that if *d*_1_ > 1.771135568, there are chaotic dynamics where the range of *q*_1_ is much smaller than that of *q*_2_. When *d*_1_ = 1.771135568, a 4-cycle orbit appears and transitions to an 8-cycle orbit as the value of *d*_1_ slightly decreases. However, the 8-cycle orbit transitions back to a 4-cycle orbit when *d*_1_ = 1.681090545, then suddenly transitions to chaos when *d*_1_ = 1.651075538. After that, when *d*_1_ = 1.339669835 and *d*_1_ = 0.918209105, 3-cycle orbits and 2-cycle orbits occur, respectively. As we reduce *d*_1_ further, these periodic solutions transform into chaotic attractors through a cascade of period-doubling bifurcations. If −0.131065533 < *d*_1_ < 0.285392696, there exists one stable equilibrium. Then, if we reduce *d*_1_ further, we discover the route to chaos through a series of period-doubling bifurcations.

**Fig 5:**
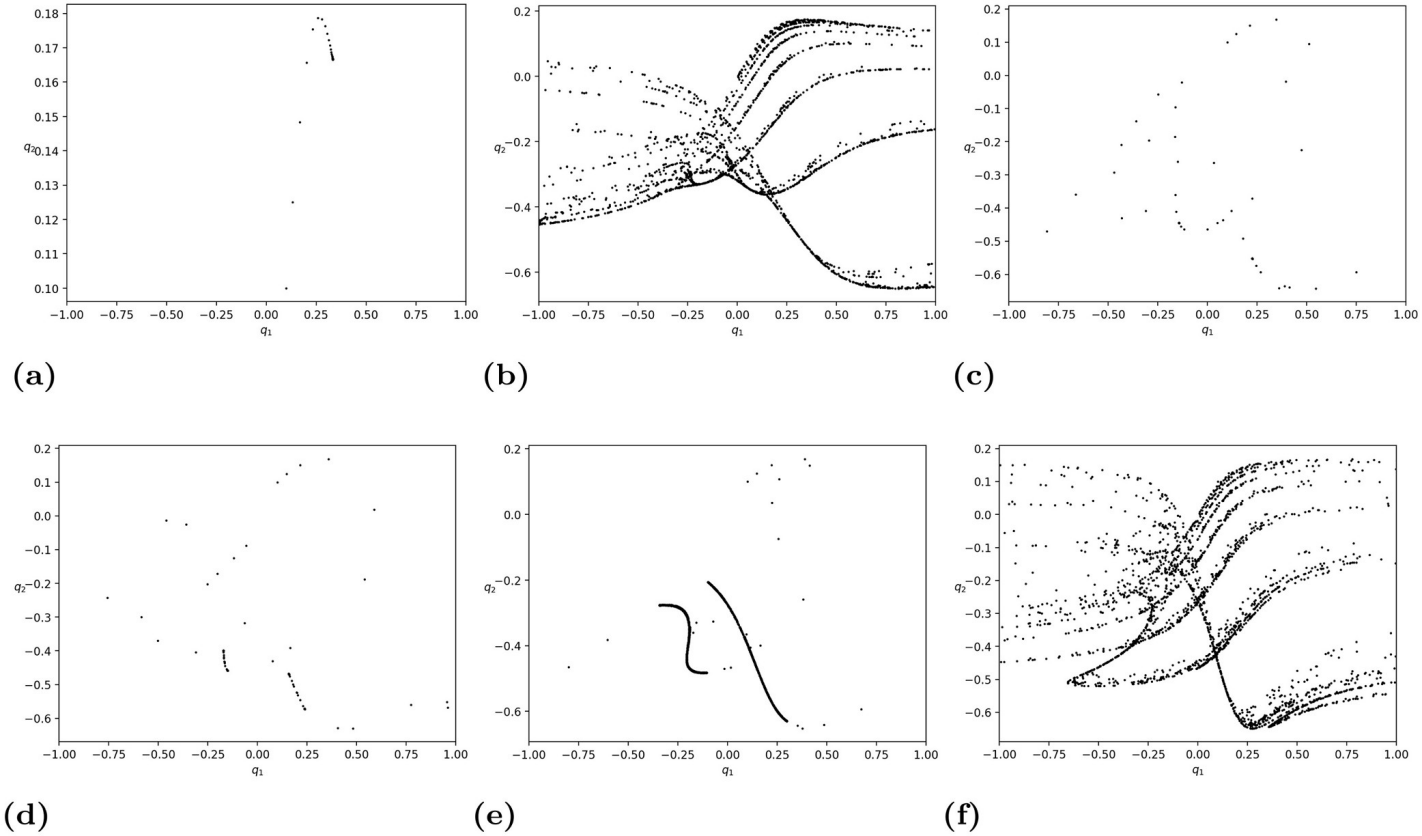
The phase portraits of map (10) by fixing the parameters *d*_2_ = 1, *c*_1_ = *c*_2_ = 1. We choose (*q*_1_(0), *q*_2_(0)) = (0.1, 0.1) to be the initial state of the iterations. (a) *d*_1_ = −0.500000. (b) *d*_1_ = −1.555110. (c) *d*_1_ = −2.700401. (d) *d*_1_ = −2.781563. (e) *d*_1_ = −2.979960. (f) *d*_1_ = −4.008016.

In Fig 5, we list some phase portraits of map (10) by fixing the parameters *d*_2_ = 1, *c*_1_ = *c*_2_ = 1, which correspond to Fig 4(b). One can see that when *d*_1_ = −0.500000, the trajectory converges to the stable equilibrium (0.33333333, 0.16666667). When *d*_1_ = −1.555110, a chaotic attractor appears with the range of *q*_2_ much larger than that of *q*_1_. When *d*_1_ = −2.700401, the trajectory starting from the initial state (*q*_1_(0), *q*_2_(0)) = (0.1, 0.1) seems to approach a periodic orbit. By further calculations, the trajectory becomes a 3-cycle as follows:

$$\begin{array}{c} \begin{array}{cc} & \left(-0.14411468,-0.44472274)\to (-8.4758964,-0.51245987\right)\\ & \to (0.22621958,-0.55196698)\to (-0.14411468,-0.44472274). \end{array} \end{array}$$

When *d*_1_ = −2.781563, a stable 6-cycle orbit exists, bifurcating from the above 3-cycle. When *d*_1_ = −2.979960, we have an attractor different from the one in Fig 5(b). However, when *d*_1_ = −4.008016, chaotic dynamics similar to Fig 5(b) reappear.

**Fig 6:**
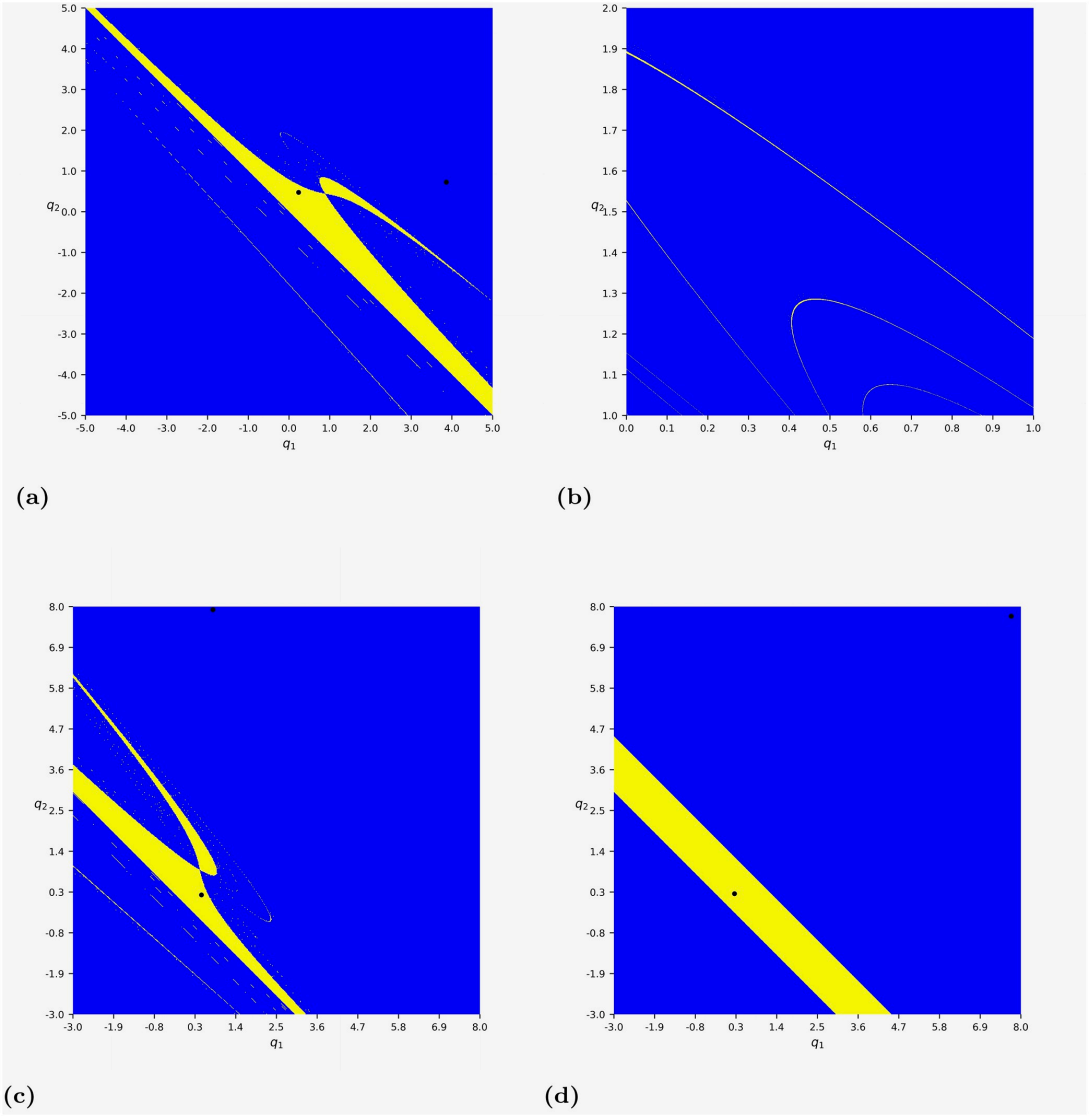
The attractors and their basins of map (10) with *c*_1_ = *c*_2_ = 1. (a) The two attractors (3.862, 0.726) and (0.235, 0.477) when (*d*_1_, *d*_2_) = (−1/8, −9/16). (b) The enlargement of (a) for (*q*_1_, *q*_2_) ∈ (0, 1) × (1, 2). (c) The two attractors (0.785, 7.917) and (0.476, 0.224) when (*d*_1_, *d*_2_) = (−73/128, −1/16). (d) The two attractors (7.742, 7.742) and (0.258, 0.258) when (*d*_1_, *d*_2_) = (−1/16, −1/16).

As mentioned in Remark 2, the multiple stable equilibria result in equilibrium selection issue. In this case, the initial state plays an important role in the long-run behavior of the model dynamics. Therefore, the selected equilibrium is path-dependent. The problem of equilibrium selection naturally leads to determining the boundaries of the basins of attraction, whose solution requires a global study of the dynamical properties of map (10). For parameter (*d*_1_, *d*_2_) = (−1/8, −9/16) in Remark 2, Fig 6(a) depicts the basins of the two stable positive equilibria in the duopoly game considered in Section 4. From a mathematical rather than economic point of view, it is interesting to explore initial points such that *q*_1_(0)<0 or *q*_2_(0)<0, thus we show the region [−5, 5] × [−5, 5] in Fig 6(a). We can see that all trajectories starting from the yellow region converge to the equilibrium (0.235, 0.477) close to the origin, while those starting from the blue region approach the other equilibrium (3.862, 0.726). The blue region is much larger than the yellow one. Furthermore, Fig 6(B) depicts the enlargement of Fig 6(a) for (*q*_1_, *q*_2_) ∈ (0, 1) × (1, 2). We can see that the topological structure of the basins of attraction is complex, which means that the outcome of the economic system sensitively depends on the initial conditions of the involved firms. In other words, a small perturbation of the initial condition may lead to crossing the boundary of the two basins of attraction. Then, the trajectory may converge to a different equilibrium. It should be emphasized that the complexity investigated here is different from the complexity we discussed in Figs 3 and 4.

Moreover, in Fig 6(c), we report the basins of attraction of the two stable positive equilibria (0.785, 7.917) and (0.476, 0.224) in map (10) with *c*_1_ = *c*_2_ = 1 and (*d*_1_, *d*_2_) = (−73/128, −1/16). A similar topological structure of the boundaries of basins can be found. In comparison, Fig 6(d) plots the basins of the two attractors (7.742, 7.742) and (0.258, 0.258) in the duopoly game of identical *d*_*i*_, i.e., *d*_1_ = *d*_2_ = −1/16. Different from the cases of (*d*_1_, *d*_2_) = (−1/8, −9/16) and (*d*_1_, *d*_2_) = (−73/128, −1/16), the boundaries of the basins of attraction are topologically simpler, which are two straight lines. Specifically, Fig 6(d) is related to Proposition 2, showing that the two equilibria with $Q_{2}^{*}>0$, $Q_{3}^{*}>0$ are both stable if *C*^2^ > −32*d*/3. For the parameters in Fig 6(d), we have that *C* = *c*_1_ + *c*_2_ = 2 and *d* = *d*_1_ = *d*_2_ = −1/16. Thus, *C*^2^ > −32*d*/3 is fulfilled, meaning there are two positive stable equilibria. As an example, Fig 6(d) gives us some hints on the possible topology of the basins of equilibria in the game of homogeneous *d*_*i*_, which may be much simpler than that of heterogeneous *d*_*i*_.

## 6 Conclusions

In this paper, we investigate the Cournot oligopoly model with an isoelastic demand function, assuming firms have quadratic cost functions. We employ the boundedly rational adjustment mechanism proposed by Bischi et al. [19] for the players, thus avoiding two unreasonable assumptions that are usually defaulted in static Cournot models: (1) the assumption that firms can anticipate their rivals’ production plans in advance, and (2) the assumption that firms know the demand function of the market. Thus, our model is closer to the real economies.

We establish a sufficient condition for the equilibrium of the model to be locally stable for the general case (see Proposition 1). In particular, we focus on the homogeneous case where firms’ marginal costs change at equal rates, i.e., identical *d*_*i*_. We obtain an analytical solution for the market supply corresponding to the Cournot-Nash equilibrium and find a sufficient condition for the equilibrium to be locally stable. Our result extends the work of Fisher [4] and that of McManus and Quandt [5], with the difference that they only consider linear demand functions, while we study nonlinear and isoelastic demand functions. Also, we find stability conditions similar to those of Fisher [4], McManus and Quandt [5]. Furthermore, we demonstrate the possibility of multiple stable equilibria under economies of scale, in contrast to the models of Fisher [4], McManus and Quandt [5] that have a unique stable equilibrium. We also illustrate that multiple equilibria may make the results of the comparative static analysis strange, thus making the model less predictive. Equilibrium selection is critical for economic models with multiple stable equilibria. Mathematically, a global analysis of discrete dynamical systems and the study of the attraction basin of each stable equilibrium is a feasible way to determine how the system will behave, but this requires complex mathematical theories and powerful computational tools.

The case of heterogeneous *d*_*i*_ is much more complex, thus we investigate the duopoly game with heterogeneous *d*_*i*_. Methods of symbolic computations such as triangular decomposition and partial cylindrical algebraic decomposition are employed in the analytical investigations of the equilibrium, which is nearly impossible by using the pencil-and-paper approach since the closed-form equilibrium is quite complicated. According to the computational results, we derive that there may exist two stable positive equilibria if both *d*_1_ and *d*_2_ take negative values. Additionally, we conduct preliminary numerical simulations and find two different types of complex dynamics of the models considered: complex trajectories such as periodic and chaotic orbits may appear in both the homogeneous and heterogeneous *d*_*i*_ cases; the topological structure of the basins of attraction may be complex in the case of heterogeneous rather than homogeneous *d*_*i*_.

In addition to the LMA adjustment mechanism used in our paper, other mechanisms of adjusting firms’ yield have been studied in the literature, such as the best response mechanism adopted by Theocharis [3], the adaptive mechanism by Fisher [4], and the myopic mechanism by Bischi et al. [15]. It is also interesting in theory to investigate the impact of other adjustment mechanisms on the local stability of the Cournot oligopoly game. Both of the above two issues deserve to be studied in depth and left to be explored in the future.

## Supporting information

S1 Appendix (PDF)

S1 Data (ZIP)

## References

[1] Cournot AA. Recherches sur les principes mathématiques de la théorie des richesses. Paris: chez L. Hachette; 1838.

[2] Dixit A. Comparative statics for oligopoly. International Economic Review. 1986;27(1):107–122. doi: 10.2307/2526609

[3] Theocharis RD. On the stability of the Cournot solution on the oligopoly problem. The Review of Economic Studies. 1960;27(2):133–134. doi: 10.2307/2296135

[4] Fisher FM. The stability of the Cournot oligopoly solution: The effects of speeds of adjustment and increasing marginal costs. The Review of Economic Studies. 1961;28(2):125–135. doi: 10.2307/2295710

[5] McManus M, Quandt RE. Comments on the stability of the Cournot oligipoly model. The Review of Economic Studies. 1961;28(2):136–139. doi: 10.2307/2295711

[6] Sato R, Nagatani K. The stability of oligopoly with conjectural variations. The Review of Economic Studies. 1967;34(4):409–416. doi: 10.2307/2296559

[7] Hadar J. Stability of oligopoly with product differentiation. The Review of Economic Studies. 1966;33(1):57–60. doi: 10.2307/2296641

[8] Zhang A, Zhang Y. Stability of a Cournot-Nash equilibrium: The multiproduct case. Journal of Mathematical Economics. 1996;26(4):441–462. doi: 10.1016/0304-4068(95)00760-1

[9] Hahn FH. The stability of the Cournot oligopoly solution. The Review of Economic Studies. 1962;29(4):329–331. doi: 10.2307/2296310

[10] Okuguchi K. The stability of the Cournot oligopoly solution: A further generalization. The Review of Economic Studies. 1964;31(2):143–146. doi: 10.2307/2296196

[11] Seade J. The stability of Cournot revisited. Journal of Economic Theory. 1980;23(1):15–27. doi: 10.1016/0022-0531(80)90028-9

[12] Al-Nowaihi A, Levine PL. The stability of the Cournot oligopoly model: A reassessment. Journal of Economic Theory. 1985;35(2):307–321. doi: 10.1016/0022-0531(85)90046-8

[13] Furth D. Stability and instability in oligopoly. Journal of Economic Theory. 1986;40(2):197–228. doi: 10.1016/0022-0531(86)90072-4

[14] Dastidar KG. Is a unique Cournot equilibrium locally stable? Games and Economic Behavior. 2000;32(2):206–218.

[15] Bischi GI, Gallegati M, Naimzada A. Symmetry-breaking bifurcations and representative firm in dynamic duopoly games. Annals of Operations Research. 1999;89:252–271. doi: 10.1023/A:1018931824853

[16] Ahmed E, Agiza HN, Hassan SZ. On modifications of Puu’s dynamical duopoly. Chaos, Solitons & Fractals. 2000;11(7):1025–1028. doi: 10.1016/S0960-0779(98)00322-1

[17] Agliari A, Gardini L, Puu T. Global bifurcations in duopoly when the Cournot point is destabilized via a subcritical Neimark bifurcation. International Game Theory Review. 2006;8(01):1–20. doi: 10.1142/S0219198906000758

[18] Puu T. On the stability of Cournot equilibrium when the number of competitors increases. Journal of Economic Behavior & Organization. 2008;66(3):445–456. doi: 10.1016/j.jebo.2006.06.010

[19] Bischi GI, Naimzada AK, Sbragia L. Oligopoly games with local monopolistic approximation. Journal of Economic Behavior & Organization. 2007;62(3):371–388. doi: 10.1016/j.jebo.2005.08.006

[20] Cavalli F, Naimzada AK. A Cournot duopoly game with heterogeneous players: Nonlinear dynamics of the gradient rule versus local monopolistic approach. Applied Mathematics and Computation. 2014;249:382–388. doi: 10.1016/j.amc.2014.10.031

[21] Cavalli F, Naimzada AK, Tramontana F. Nonlinear dynamics and global analysis of a heterogeneous Cournot duopoly with a local monopolistic approach versus a gradient rule with endogenous reactivity. Communications in Nonlinear Science and Numerical Simulation. 2015;23(1-3):245–262. doi: 10.1016/j.cnsns.2014.11.013

[22] Tramontana F, Elsadany AA, Xin B, Agiza HN. Local stability of the Cournot solution with increasing heterogeneous competitors. Nonlinear Analysis: Real World Applications. 2015;26:150–160.

[23] Li X, Su L. A heterogeneous duopoly game under an isoelastic demand and diseconomies of scale. Fractal and Fractional. 2022;6(8):459. doi: 10.3390/fractalfract6080459

[24] Li X, Mou C, Wang D. Decomposing polynomial sets into simple sets over finite fields: The zero-dimensional case. Computers & Mathematics with Applications. 2010;60(11):2983–2997. doi: 10.1016/j.camwa.2010.09.059

[25] Li X, Wang D. Computing equilibria of semi-algebraic economies using triangular decomposition and real solution classification. Journal of Mathematical Economics. 2014;54:48–58. doi: 10.1016/j.jmateco.2014.08.007

[26] Li X, Li B, Liu L. Stability and dynamic behaviors of a limited monopoly with a gradient adjustment mechanism. Chaos, Solitons & Fractals. 2023;168:113106. doi: 10.1016/j.chaos.2023.113106

[27] Agiza HN. On the analysis of stability, bifurcation, chaos and chaos control of Kopel map. Chaos, Solitons & Fractals. 1999;10(11):1909–1916. doi: 10.1016/S0960-0779(98)00210-0

[28] Davis D. Behavioral convergence properties of Cournot and Bertrand markets: An experimental analysis. Journal of Economic Behavior & Organization. 2011;80(3):443–458. doi: 10.1016/j.jebo.2011.04.008

[29] Kukushkin NS. Cournot tatonnement and potentials. Journal of Mathematical Economics. 2015;59:117–127. doi: 10.1016/j.jmateco.2015.06.005

[30] Ma J, Guo Z. The influence of information on the stability of a dynamic Bertrand game. Communications in Nonlinear Science and Numerical Simulation. 2016;30(1-3):32–44. doi: 10.1016/j.cnsns.2015.06.004

[31] Zhou W, Wang X. On the stability and multistability in a duopoly game with R&D spillover and price competition. Discrete Dynamics in Nature and Society. 2019;2019:1–20. doi: 10.1155/2019/2369898

[32] Xin B, Peng W, Kwon Y. A discrete fractional-order Cournot duopoly game. Physica A: Statistical Mechanics and its Applications. 2020;558:124993. doi: 10.1016/j.physa.2020.124993

[33] Puu T. Chaos in duopoly pricing. Chaos, Solitons & Fractals. 1991;1(6):573–581. doi: 10.1016/0960-0779(91)90045-B

[34] Tuinstra J. A price adjustment process in a model of monopolistic competition. International Game Theory Review. 2004;06(03):417–442. doi: 10.1142/S0219198904000289

[35] Silvestre J. A model of general equilibrium with monopolistic behavior. Journal of Economic theory. 1977;16(2):425–442. doi: 10.1016/0022-0531(77)90017-5

[36] Aubry P, Moreno Maza M. Triangular sets for solving polynomial systems: a comparative implementation of four methods. Journal of Symbolic Computation. 1999;28(1–2):125–154. doi: 10.1006/jsco.1999.0270

[37] Kalkbrener M. A generalized Euclidean algorithm for computing triangular representations of algebraic varieties. Journal of Symbolic Computation. 1993;15(2):143–167. doi: 10.1006/jsco.1993.1011

[38] Wang D. Computing triangular systems and regular systems. Journal of Symbolic Computation. 2000;30(2):221–236. doi: 10.1006/jsco.1999.0355

[39] Wu WT. Basic principles of mechanical theorem proving in elementary geometries. Journal of Automated Reasoning. 1986;2(3):221–252. doi: 10.1007/BF02328447

[40] Collins GE, Hong H. Partial Cylindrical Algebraic Decomposition for Quantifier Elimination. Journal of Symbolic Computation. 1991;12(3):299–328. doi: 10.1016/S0747-7171(08)80152-6

