# Supplementary material for: Dynamics of a Cournot game with bounded rational firms and various scale effects
Source: PLoS One. 2024 May 28;19(5):e0297275. doi: 10.1371/journal.pone.0297275 (PMC11132496; doi:10.1371/journal.pone.0297275)
Supplement: S1 Appendix — (PDF) [file pone.0297275.s001.pdf]

Appendix

$$
\begin{aligned}
T_2 =& (2\,c_1^2 d_2^2 + d_1 c_2 c_1 d_2 + c_1 c_2 d_2^2 + 2\,d_1^2 d_2 + 4\,d_1 d_2^2 + 2\,d_2^3) q_2^* + (-4\,d_1^4 + 4\,d_1^3 d_2 \\
& + 4\,d_1^2 d_2^2 - 4\,d_1 d_2^3) q_1^{*3} + (-4\,c_1 d_1^3 - 2\,c_1 d_1^2 d_2 + 8\,c_1 d_1 d_2^2 - 2\,d_2^3 c_1 - 4\,d_1^3 c_2 \\
& + 4\,c_2 d_1 d_2^2) q_1^{*2} + (-c_1^2 d_1^2 - 2\,c_1^2 d_1 d_2 + 3\,c_1^2 d_2^2 - 2\,d_1^2 c_2 c_1 - 2\,d_1 c_2 c_1 d_2 + 2\,c_1 c_2 d_2^2 \\
& - d_1^2 c_2^2 - c_2^2 d_1 d_2 - 4\,d_1^2 d_2 - 4\,d_1 d_2^2) q_1^* - 2\,c_1 d_2^2 + c_2\,d_1^2 + c_2\,d_1 d_2. \\
T_1 =& (8\,d_1^3 - 16\,d_1^2 d_2 + 8\,d_1 d_2^2) q_1^{*4} + (12\,c_1 d_1^2 - 16\,c_1 d_1 d_2 + 4\,c_1 d_2^2 + 8\,c_2 d_1^2 - 8\,c_2 d_1 d_2) \\
& q_1^{*3} + (6\,c_1^2 d_1 - 4\,c_1^2 d_2 + 8\,d_1 c_2 c_1 - 4\,c_1 c_2 d_2 + 2\,d_1 c_2^2 + 16\,d_1 d_2) q_1^{*2} + (c_1^3 + 2\,c_1^2 c_2 \\
& + c_1\,c_2^2 + 8\,c_1 d_2 - 2\,d_1 c_2 + 2\,d_2 c_2) q_1^* - c_1 c_2 - 2\,d_2. \\
BP_1 =& d_2\,d_1 (d_1 - d_2)(d_1 + 1/2)(d_2 + 1/2)(d_1^2 + 2\,d_1 d_2 + d_2^2 + 1/2\,d_1 + 3/2\,d_2) \\
& (1024/27\,d_2^3 d_1^3 + 256/9\,d_1^3 d_2^2 + 256/9\,d_1^2 d_2^3 + d_1^4 + 28/9\,d_1^3 d_2 + 182/9\,d_1^2 d_2^2 \\
& + 28/9\,d_1 d_2^3 + d_2^4 + 16/27\,d_1^3 + 16/9\,d_1^2 d_2 + 16/9\,d_1 d_2^2 + 16/27\,d_2^3). \\
BP_2 =& d_2\,d_1 (d_1 - d_2)(d_2 + 1/2)(d_1 + 1/2)(d_1 d_2 + 1/4\,d_1 + 1/4\,d_2 + 3/64)(d_1^3 - 8\,d_1^2 d_2 \\
& + 16\,d_1 d_2^2 + 4\,d_2^2)(d_1^2 + 2\,d_1 d_2 + d_2^2 + 1/2\,d_1 + 3/2\,d_2)(d_1^2 d_2 - 1/2\,d_1 d_2^2 + 1/16\,d_2^3 \\
& + 1/4\,d_1^2)(1024/27\,d_2^3 d_1^3 + 256/9\,d_1^3 d_2^2 + 256/9\,d_1^2 d_2^3 + d_1^4 + 28/9\,d_1^3 d_2 \\
& + 182/9\,d_1^2 d_2^2 + 28/9\,d_1 d_2^3 + d_2^4 + 16/27\,d_1^3 + 16/9\,d_1^2 d_2 + 16/9\,d_1 d_2^2 \\
& + 16/27\,d_2^3)(d_1^5 d_2 - 4\,d_1^4 d_2^2 + 6\,d_2^3 d_1^3 - 4\,d_1^2 d_2^4 + d_1\,d_2^5 + 1/8\,d_1^5 - 15/8\,d_2 d_1^4 \\
& + 23/4\,d_1^3 d_2^2 + 23/4\,d_2^3 d_1^2 - 15/8\,d_2^4 d_1 + 1/8\,d_2^5 - 201/1024\,d_1^4 + 393/256\,d_1^3 d_2 \\
& + 1701/512\,d_2^2 d_1^2 + 393/256\,d_2^3 d_1 - 201/1024\,d_2^4 + 9/64\,d_1^3 + 27/64\,d_1^2 d_2 \\
& + 27/64\,d_1 d_2^2 + 9/64\,d_2^3).
\end{aligned}
$$
